# O-Glycome Beam Search Arrays for Carbohydrate Ligand Discovery*[Fn FN2]

**DOI:** 10.1074/mcp.RA117.000285

**Published:** 2017-11-28

**Authors:** Zhen Li, Chao Gao, Yibing Zhang, Angelina S. Palma, Robert A. Childs, Lisete M. Silva, Yang Liu, Xi Jiang, Yan Liu, Wengang Chai, Ten Feizi

**Affiliations:** From the ‡Glycosciences Laboratory, Department of Medicine, Imperial College London, W12 0NN, UK;; §Department of Surgery, Beth Israel Deaconess Medical Center, Harvard Medical School, Boston, Massachusetts 02215;; ¶Department of Chemistry, UCIBIO-NOVA University of Lisbon, 1099085, Portugal,; ‖Division of Infectious Diseases, Cincinnati Children's Hospital Medical Center and; **University of Cincinnati College of Medicine, Cincinnati, Ohio 45229

## Abstract

*O*-glycosylation is a post-translational modification of proteins crucial to molecular mechanisms in health and disease. *O*-glycans are typically highly heterogeneous. The involvement of specific *O*-glycan sequences in many bio-recognition systems is yet to be determined because of a lack of efficient methodologies. We describe here a targeted microarray approach: *O*-glycome *beam search* that is both robust and efficient for *O*-glycan ligand-discovery. Substantial simplification of the complex *O*-glycome profile and facile chromatographic resolution is achieved by arraying *O*-glycans as branches, monitoring by mass spectrometry, focusing on promising fractions, and on-array immuno-sequencing. This is orders of magnitude more sensitive than traditional methods. We have applied beam search approach to porcine stomach mucin and identified extremely minor components previously undetected within the *O*-glycome of this mucin that are ligands for the adhesive proteins of two rotaviruses. The approach is applicable to *O*-glycome recognition studies in a wide range of biological settings to give insights into glycan recognition structures in natural microenvironments.

Mucins are glycoproteins crucial in molecular mechanisms in health and disease (1–3). They occur in secretions and in membrane-associated forms on epithelia, endothelia and leukocytes being associated with tissue- and cell-specific activities such as cell-attachment of commensal and pathogenic microbes, targeting of leukocytes to endothelial cells and modulation of immune responses (4). The glycans on mucins are *O*-glycosidically linked to serine or threonine, and range from a single to 20 or more monosaccharide residues (5). The *O*-glycans are known to express species- and tissue-specific antigens that change during cell differentiation and oncogenesis (2, 6), and mediate some of the above-mentioned mucin functions (7). However, the involvements of specific *O*-glycan structures in many processes are yet to be determined (2), assignments being hampered by the diversity of extensions at the core-monosaccharide, GalNAc, and multiplicity of chain lengths and branching patterns (5, 8, 9), and often the limited amounts of glycans available. Thus high-throughput and microscale methodologies are needed for deconvolution and functional assignments of individual *O*-glycans within *O*-glycomes as cues to large scale syntheses of desired glycan sequences for exploitation (10, 11).

In classical studies, *O*-glycans released from abundantly available mucins (from ovarian cystadenomas) were used to elucidate the structures of the major blood group antigens A, B, H and Lewis^a^ (Le^a^) and Le^b^ (12, 13). *O*-glycans or their fragments were used as inhibitors of antibody binding requiring large amounts (milligrams) of individually purified glycans. To address the need for a microscale method for determining recognition of individual glycans by antibodies and other carbohydrate-binding proteins, the neoglycolipid (NGL)^1^ technology was introduced whereby glycans, singly or as mixtures, are conjugated to a lipid molecule, and immobilized on solid matrices, resolved by thin layer chromatography (TLC) and probed with carbohydrate-binding proteins (14–16). The NGL technology coupled with mass spectrometry has undergone sequential developments (17, 18), and has been the basis of the first microarray system for sequence-defined glycans (19). This is now a state-of-the-art platform with the glycan probes robotically arrayed in a liposomal formulation at low femtomoles per spot (20, 21). A “designer” array of NGLs from an epithelial *O*-glycome, resolved on TLCs enabled the characterization of the elusive prostate cancer-associated antigen F77 (22).

The set of microscale methods described here for *O*-glycan populations (Fig. 1) is a significant advance involving, as an early step, microarray analysis of NGL populations resolved by HPLC with mass spectrometry (MS) monitoring rather than TLC plate binding analyses (commonly referred to as chromatogram binding), which necessitates elution of bound components (22). There follows iterative microarray analyses of targeted ligand-positive fractions resolved by appropriate chromatographic separations concomitantly with MS and culminating in micro-immuno-sequencing of a ligand-positive glycan. We coin the term beam search because the power to search for and focus on ligand-positive components in a population of hundreds of *O*-glycans within an epithelial *O*-glycome. As an exemplar study-case, applying the approach to a ligand-bearing mucin, we identify *O*-glycan ligands for the cell-adhesion proteins, VP8*, of two rotaviruses P[19] and P[10]. These are evolutionarily closely related but of distinct genotypes. The first, infects humans and pigs, and the second has so far been reported to infect humans. Like VP8* proteins of other rotaviruses, those of P[10] and P[19] bind human salivary mucins and porcine stomach mucin (PSM), but the glycan ligands in these have not been characterized (23, 24). The approach is complemented by microarrays of sequence-defined glycans.

**Fig. 1.**
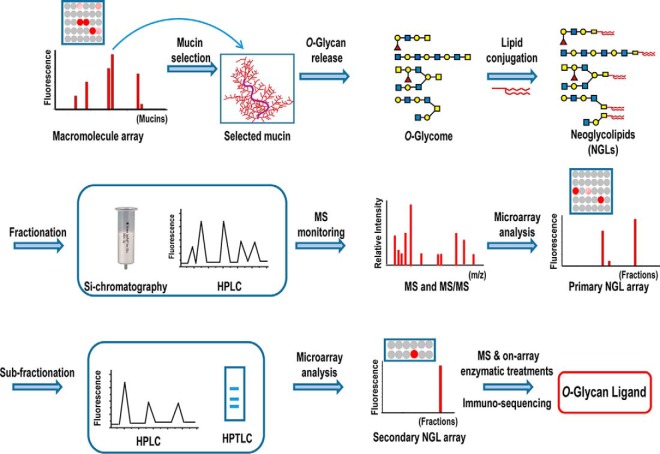
**Scheme for the *O*-glycome beam search approach to target naturally occurring ligands within heterogeneous populations.** The macromolecule array is of mucins. A ligand-bearing mucin is selected after microarray analyses of a carbohydrate recognizing protein. *O*-glycan alditols released from the mucin are converted, after mild periodate oxidation, to fluorescent neoglycolipids (NGLs) derived from branches 3- or 6-linked to core *N*-acetylgalactosaminitol with resulting marked simplification of glycan profiles. The NGLs are fractionated by chromatographies such as silica chromatography and HPLC, monitored by mass spectrometry (MS) and robotically arrayed. These fractions constitute the primary array. Microarray analyses using the primary array enable targeting of ligand-positive fractions. These are sub-fractionated as necessary with iterative secondary or tertiary microarray analyses to identify, isolate and characterize the ligands by MS and various microscale structural analyses including on-array enzymatic treatments and immuno-sequencing. Symbol Nomenclature for Glycans (SNFG) is used for monosaccharides: 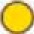, galactose; 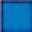, N-acetylglucosamine; 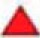, fucose; 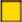, *N*-acetylgalactosamine. For simplicity the modification of the core *N*-acetylgalactosaminitol is not shown.

## EXPERIMENTAL PROCEDURES

### 

#### 

##### Recombinant VP8* Proteins of P[10] and P[19] Rotaviruses

The recombinant rotavirus VP8* proteins were expressed as glutathione S-transferase (GST)-tagged proteins as described (25).

##### Monoclonal Anti-blood group Antibodies (MAbs) and Plant Lectins

Murine anti-A type 1 IgM (AH21), anti-A Lewis^b^ IgG (HH3) were gifts from Henrik Clausen, Center for Glycomics, University of Copenhagen. Anti-A type 2 IgM (Z2A), anti-A IgG (T36), anti-H type 1 IgG (17–206), and anti-H type 2 IgG (BRIC231), were from Abcam (Cambridge, UK). Anti-lacto-*N*-tetraose (LNT) IgM (SIG-3310) was from Signet Laboratories (Hayward, CA). Biotinylated lectins *Ulex europaeus* agglutinin I (UEA-I) and *Aleuria aurantia* lectin (AAL), were from Vector Laboratories (Peterborough, UK).

##### Mucins and Mucin-enriched Glycoproteins

Porcine stomach mucin (PSM) was from Sigma (Dorset, UK); other mucins and mucin-enriched glycoproteins in supplemental Table S1 have been described previously (22).

##### O-Glycans

Steps in the release and processing of PSM *O*-glycome and derived NGL fractions for robotic arraying are in supplemental Fig. S1. *O*-Glycans were released from PSM, 1 g dry weight, by reductive alkaline borohydride degradation (22) and fractionated initially by anion-exchange chromatography on an AG1X8 column (Bio-Rad, Hercules, CA, acetate form, 3 ml resin), with sequential elution with 2 mM and 300 mM pyridium acetate, followed by 300 mM Na_2_SO_4_ to yield predominantly neutral, sialylated and sulfated fractions, respectively. The neutral fraction was the subject of the present study. The neutral fraction was chromatographed on a Bio-Gel P4 (Bio-Rad) column (1.6 × 90 cm) eluted with water at a flow rate of 15 ml/h. Fractions were monitored on-line by refractive index and their carbohydrate contents with respect to hexose were determined by the dot orcinol-sulfuric acid method using galactose as standard (18).

##### NGLs

Fluorescent NGLs were prepared from the reductively released oligosaccharides (alditols) using *N*-aminoacetyl-*N*-(9-anthracenylmethyl)-1,2-dihexadecyl-sn-glycero-3-phosphoethanolamine (ADHP) after mild periodate oxidation (18). In brief, lyophilized *O*-glycan fractions from the Bio-Gel P4 column (∼1 μmol of each, estimated based on the average molecular weights and the sugar contents in the factions as in supplemental Table S2) were mixed with freshly prepared sodium periodate (700 μl, 1.25 mg/ml in imidazole buffer, pH 6.5). Reaction mixtures were kept on ice in the dark for 5 min followed by addition of 80 μl butane-2,3-diol (9 mg/ml) and incubation in the dark for a further 40 min. ADHP (2 ml of 10 mm solution in CHCl_3_/MeOH, 1:1 by volume) and 1.45 ml freshly prepared tetrabutylammonium cyanoborohydride (20 mg/ml in MeOH) were then added, and the reaction carried out at 60 °C for 16–24 h. Reaction mixtures were analyzed by HPTLC developed with CHCl_3_/MeOH/H_2_O, 60:35:8, and visualized under long-wave length UV light. NGLs were quantified with respect to their fluorescent lipid contents by spectrophotometry at 368.4 nm using ADHP as a standard (22).

A glycolipid and 11 NGLs (18, 20, 22) were included as reference compounds in the tertiary array designated “Fucase Array” (see below). Their sequences are given in supplemental Table S6. These included H2, LNFP-I.DH (lacto-*N*-fucopentaose I), LNnFP-I.DH (lacto-*N*-neofucopentaose I), GSC-915–3.DH, GSC-915–4. pLNH.DH (*p*-Lacto-*N*-hexaose), pLNH-b.DH, pLNnH.DH (*p*-Lacto-*N*–neohexaose), LNDFH-I.DH (Lacto-*N*-difucohexaose I), LNFP-II.DH (Lacto-*N*-fucopentaose II) (22, 26).

##### High-performance Liquid Chromatography (HPLC)

NGLs were fractionated by normal phase HPLC with a XBridge Amide column (3.5 μm, 4.6 × 250 mm, Waters, Borehamwood, UK) using a Gilson system coupled with a fluorescence detector (λ_ex_ at 255 nm and λ_em_ at 405 nm) (22). The gradient was CHCl_3_/MeOH/H_2_O 130:70:9 (solvent A) to 10:20:8 (solvent B) in 60 min at a flow rate of 0.5 ml/min.

##### Semipreparative High Performance Thin Layer Chromatography (HPTLC)

For semi-preparative TLC, NGLs were dissolved in CHCl_3_/MeOH/H_2_O, 25:25:8 and applied on the aluminum backed HPTLC plates (Merck, Darmstadt, Germany), and were chromatographed in CHCl_3_/MeOH/H_2_O, 60:35:8. For improved separation, the plate was dried and developed in the same solvent for a second time. The NGLs were visualized under 254 nm UV light. The TLC bands of interest were scraped off the plates, and the NGLs eluted with CHCl_3_/MeOH/H_2_O, 25:25:8 from a mini-column packed with the silica gel.

##### Mass Spectrometry

Negative-ion electrospray-ionization MS (ESI-MS) and electrospray ionization collision-induced MS/MS (ESI-CID-MS/MS) analyses of oligosaccharides were carried out on a Waters Synapt G2-S instrument. Oligosaccharides were dissolved in water at 5–10 pmol/μl and 1–2 μl was loop-injected for analysis. ACN/H_2_O (1:1) containing 5 mm NH_4_HCO_3_ was used as the elution solvent. Cone voltage 80 V and the collision gas Ar with collision energies of 15–30 V were used for CID-MS/MS. Matrix-assisted laser desorption ionization (MALDI-MS) and MALDI-MS/MS analyses of NGLs were carried out in both negative- and positive-ion mode on Shimadzu AXIMA Assurance (linear TOF) and Resonance (QIT-TOF) instrument, respectively. Oligosaccharides and NGLs were dissolved in water and CHCl_3_/MeOH/H_2_O 25:25:8, respectively, at a concentration of 10–20 pmol/μl, and 0.5 μl was deposited on the sample target together with 1 μl of matrix: 2′,4′,6′-trihydroxyacetophenon. Ethanol was used for recrystallization. For CID-MS/MS, a collision gas Ar (2 bar) and optimized collision energy in the region of 80–140 V were used to obtain fragmentation.

##### Glycan Microarrays

Five microarrays on 16-pad nitrocellulose-coated glass slides (Sartorius Stedim, Goettingen, Germany) using a noncontact arrayer, Nano-Plotter (Gesim, Germany) were used in the present study. These included (a) the macromolecule array of mucin-rich glycoproteins; (b) the primary NGL array of 60 fractions of the PSM neutral *O*-glycome resolved by HPLC (supplemental Table S4); (c) the secondary NGL array designated Fucase Array (supplemental Table S6) which was generated for on-array fucosidase digestion experiments and consisted of NGLs of four PSM *O*-glycan subfractions and 12 sequence-defined lipid-linked glycan probes; and (d) a screening array of sequence-defined glycans designated “F77/Ii array” consisting of 60 lipid-linked glycan probes (supplemental Table S7), in part described previously (19), which was used in the validation of the beam search results; and (e) a small array of fungus-derived gluco oligosaccharides (supplemental Table S8).

The macromolecule array was of 30 mucin-enriched epithelial glycoproteins designated “Mucin Array” (22), which consisted of extracts from ovarian cystadenoma fluids, meconia, several purified mucins of human origin and two of animal origins (PSM and BSM). Two array sets of these were used: mucin set 2 was used in the analyses of the VP8* proteins in 2. 2A and 2B, and mucin set 3 in analyses of the antibodies and lectins in supplemental Table S1 and supplemental Fig. S2. These were arrayed in water (22) at 30 and 170 pg hexose per spot. Binding signals were glycoprotein dose-related; results at 170 pg hexose per spot level are shown.

The primary and secondary NGL arrays, and the “F77/Ii array” of lipid-linked glycan probes (NGLs and glycolipids) were generated in a liposomal formulation (20). In brief the printing solutions contained 100 pmol/μl of phosphatidylcholine and cholesterol (both from SIGMA) as lipid carriers in addition to the lipid-linked glycan probes in water (HPLC grade). The concentrations of the lipid-linked glycan probes were 5 and 15 pmol/μl for the 2 and 5 fmol per spot levels, respectively. The printing solutions also contained Cy3 NHS ester (GE Healthcare, Buckinghamshire, UK) at 20 ng/ml (26 fmol/μl) as a marker to monitor the printing process.

Microarray binding analyses were performed at ambient temperature, essentially as described (20). In brief, for analyses of the viral proteins, the arrayed slides were blocked for 1 h with 0.02% (w/v) casein (Pierce, ThermoFischer Scientific, Walham, MA) and 1% bovine serum albumin (BSA, Sigma) in HBS (5 mm HEPES buffer pH 7.4, 150 mm NaCl) with 5 mm CaCl_2_ (Blocker A). The VP8* proteins of P[10] and P[19] viruses were diluted to 25 μg/ml, 50 μg/ml or 100 μg/ml, as indicated, in Blocker A and overlaid for 1.5 h, followed detection for 1 h with rabbit anti-GST polyclonal antibody (Santa Cruz, Dallas, TX) and biotinylated anti-rabbit IgG (Sigma), both at 1:200 in Blocker A. For analyses of the mAbs, the arrayed slides were blocked with 3% BSA in HBS with 5 mm CaCl_2_ (Blocker B) and overlaid with the anti-H type 1 at 1:50 dilution; anti-H type 2, anti-A, anti-LNT, all at 1:100 dilution; anti-A type 2 at 1:200 dilution; anti-A type 1 and anti-A Lewis^b^ culture supernatant undiluted. Binding was detected with biotinylated anti-mouse IgG (Sigma) at 1:200 dilution or anti-mouse IgM (Sigma, μ-chain-specific) at 1:500 dilution. The biotinylated plant lectin UEA-I were analyzed using a single step overlay at 50 μg/ml. For mAbs and lectins Blocker B was used as diluent. The final detection step was carried out by 30 min overlay with Alexa Fluor-647-labeled streptavidin (Molecular Probes, Eugene, OR, 1 μg/ml in the corresponding blockers above).

Fluorescence images were recorded using ProScanArray microarray scanner (PerkinElmer LAS, Beaconsfield, UK) or GenePix 4300A (Molecular Devices, Sunnyvale, CA). For both scanners scanning resolution of 10 μm/pixel was used and this resolution was adequate for the sizes of sample spots. Red laser (scan wavelength 633 nm) was used to record the protein binding signals. Images were analyzed using ScanArray Express software (PerkinElmer, Beaconsfield, UK) or GenePix® Pro 7 Microarray Analysis software (Molecular Devices, Workingham, UK), and the quantified gpr file was entered into an in-house microarray database using dedicated software (27) for data processing. No normalization method or statistical analysis was used for data processing, nor software or algorithms used for interpretation of processed data. The binding signals were dose related. Results are given for binding signals at 5 fmol per spot.

The above information is compliant with the MIRAGE (Minimum Information Required for A Glycomics Experiment) guidelines for reporting glycan microarray-based data (28) (SI Appendix).

##### On-array Fucosidase Digestion

For on-array glycosidase digestion, the array was hydrated with HEPES-buffered saline (HBS, 5 mm HEPES, pH 7.4, 150 mm NaCl, 5 mm CaCl_2_) for 2 min and blocked with 3% bovine serum albumin (BSA, Sigma) in HBS buffer for 1 h before addition of α1,2-fucosidase (New England Biolabs, Hitchin, UK, P0724L Lot 0111505) in 50 mm sodium citrate buffer, supplemented with 100 mm NaCl and 1 μg/ml BSA (pH 6.0). The enzyme was evaluated at 8–20 unit/μl and incubation was at 37 °C for 12–44 h. The binding analyses were performed after treatment with the enzyme at 8–20 unit/μl. The array was then washed 3 times with HBS and once with water and dried at ambient.

## RESULTS

### 

#### 

##### Macromolecule Array for Selection of a Ligand-bearing Mucin Glycoprotein

**Fig. 2.**
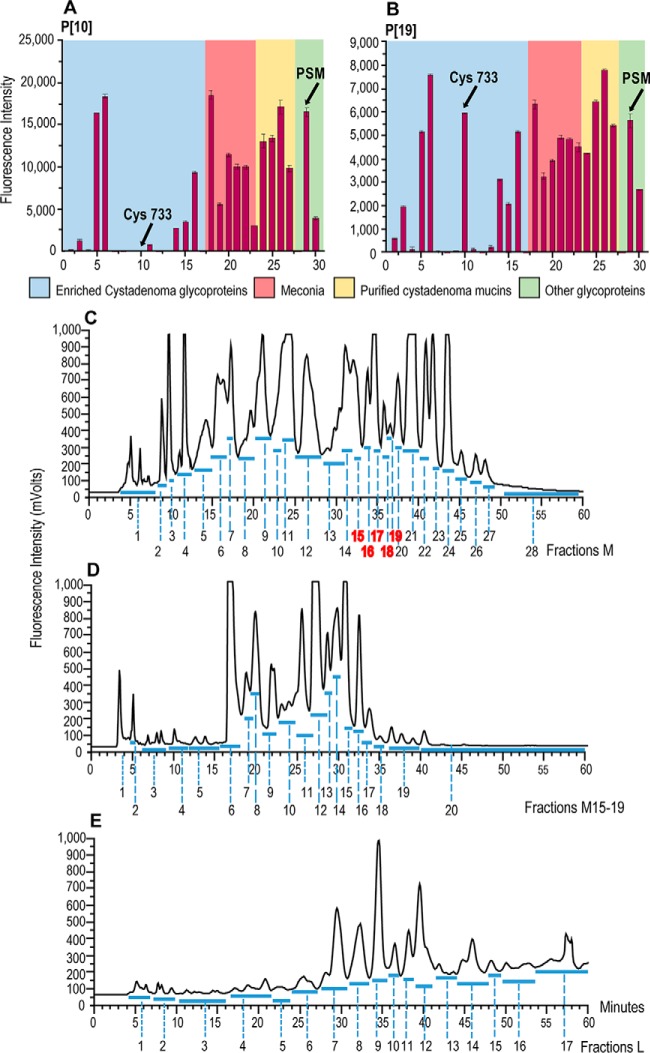
**Steps in the preparation of the *O*-glycome beam search array.** (*A*) and (*B*) Microarray analyses of the VP8* proteins of P[10] and P[19] enteroviruses at 25 μg/ml,with a panel of 30 mucin-enriched glycoproteins (macromolecule array). *C–E*, Fractionation by HPLC of neutral *O*-glycome NGLs derived from PSM *O*-glycan alditols; the NGLs with *m*iddle migration patterns M, (described in supplemental Fig. S5) are in (*C*) and fractions are designated MHP1-MHP28; re-fractionation of the pooled fractions MHP15–19 is in (*D*) and the sub-fractions are designated ^M15–19^HP1- ^M15–19^HP20; the NGLs with *l*ower migration patterns, L, are in (*E*) and fractions are designated LHP1-LHP17. These NGL fractions constitute the primary array.

The work flow in the beam search approach (supplemental Fig. S1) began with the use of a microarray of 30 mucin-enriched glycoprotein preparations in order to select, as a study-case, a potent ligand-bearing mucin to reveal natural ligands therein for the VP8* proteins of rotaviruses P[10] and P[19]. Among the mucins were those derived from human ovarian cystadenomas, meconia, and porcine stomach mucin (PSM) and bovine submaxillary mucin (BSM) (supplemental Table S1). There was binding by both VP8* proteins to preparations of all five of the meconium-derived, eleven of the cystadenoma-derived mucins as well as to the PSM and BSM (Fig. 2*A* and 2*B* and supplemental Fig. S2). The binding patterns of the two viral proteins were similar overall, except that the P[19] VP8* but not the P[10] VP8* bound strongly to one additional cyst mucin, Cyst 733 (position 10, Fig. 2A and 2B, supplemental Table S1 and supplemental Fig. S2). Among the ligand-positive mucins, we selected PSM for the *O*-glycome beam search for ligands for the P[10] and P[19] VP8* proteins.

##### Preparation of O-glycome and NGL Probes

*O*-Glycans were reductively released from PSM, 1 g dry weight, and fractionated by gel filtration chromatography on a column of Bio-Gel P4 (supplemental Fig. S3). Thirteen pooled fractions (a to m) were collected. To have an overview of the *O*-glycan compositions the fractions were analyzed by MALDI-MS. Fractions a, b, c, l and m contained little or no detectable carbohydrate as indicated by MS-analyses and were not investigated further. In fractions d to k (total hexose content: 42 mg estimated to be equivalent to 84 mg sugar, considering that *N*-acetylhexosamines give no response in hexose assay (29)), *O*-glycan alditols with twenty-three molecular ions were detectable as major components, corresponding to sizes ranging from trisaccharide in fraction k to hexadecasaccharide in fraction d (supplemental Table S2). Undoubtedly there would be isomeric glycan species among these, as well as a multitude of additional minor components not detectable within the eight fractions under the MS conditions used.

Around 1 mol of each of the *O*-glycan alditol fractions d to k (estimate based on average molecular weights in the factions; total sugar about 10 mg) was used for conversion to fluorescent NGLs (17) after mild periodate oxidation (18), and these were analyzed by high performance (HP) TLC (supplemental Fig. S4). The oxidative splitting of the core GalNAcol between C4 and C5 gives rise to two reactive aldehydes; one bearing the 6-linked glycan branch (if present) and the other 3-linked branch at GalNAcol (18) (supplemental Scheme S1). The monosaccharide compositions of the main NGL components detected in each of the fractions d to k are deduced from MALDI-MS analyses. At least twenty-three 3OX- molecular ions ranging from mono- to decasaccharides, and eleven 6OY-NGLs molecular ions ranging from mono- to hexasaccharides could be detected (supplemental Table S3).

The number of the 3OX-NGLs derived from each of the fractions (d to k) ranged from six to seventeen; the 6OY-NGLs were fewer, and ranged from four to eight. There were 3OX- and 6OY-NGL species with the same monosaccharide compositions in the eight fractions (unrelated to the sizes of the original *O*-glycan alditols). These represent chains 3-linked to core GalNAcol that may be accompanied by one or other of the 6-linked chains (supplemental Table S3). All of the fractions contained NGLs with the compositions H_1_-3OX, H_1_.N_1_-3OX and H_1_.N_1_-6OY; each of fractions f, g, i, j and k contained N_1_-6OY; and each of the fractions d, e, i, j and k contained dH_1_.H_2_.N_1_-3OX. These findings directed us to pool the NGLs of fractions d to k and thereby have similar molecules in one pot rather than in different fractions. The pool was fractionated and excess lipid reagent was removed by a small silica column (supplemental Fig. S5). NGL fractions with middle (M) and lower (L) migrations on TLC, which contain tri- to nonasaccharides and hexa- to undecasaccharides oligosaccharides, respectively were combined and subjected to HPLC fractionation. The upper fraction which contained NGLs ranging from mono- to tri-saccharides was not pursued in present study as in the exploratory beam search it was found to be ligand-negative.

##### Primary NGL Microarray to Identify Ligand-positive O-Glycome Fractions

**Fig. 3.**
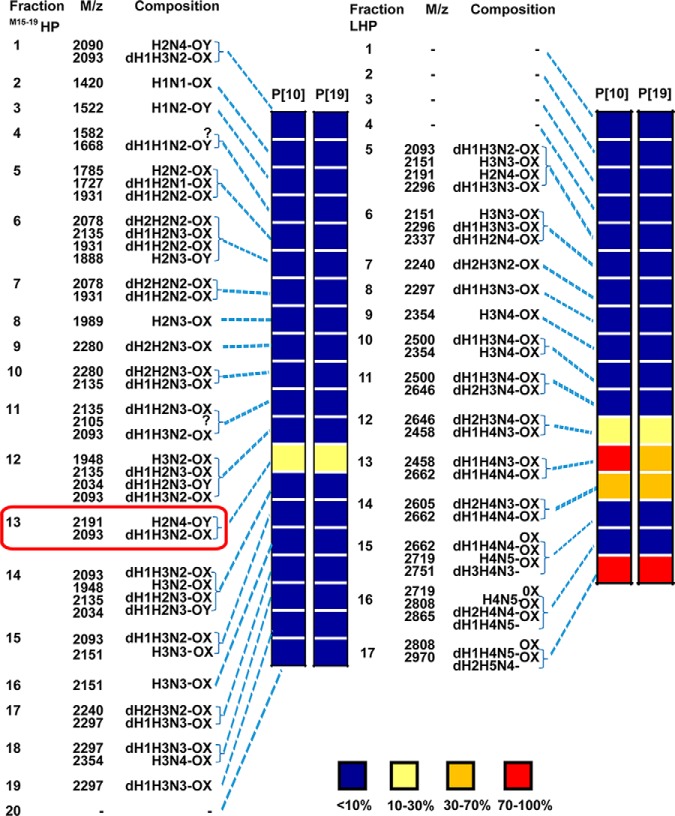
Relative intensities of fluorescence in microarray analyses of the VP8* proteins of rotaviruses P[10] and P[19] analyzed at 50 μg/ml) with neutral *O*-glycome NGL fractions ^M15–19^HP1 to ^M15–19^HP20 and LHP1 to LHP17. The molecular ions in MALDI-MS and deduced monosaccharide compositions of the fractions are also given. Inset, relative intensities are given with 100% for the highest signal for each protein. The fluorescence values for all of the arrayed neutral *O*-glycome NGL fractions that constitute the secondary array are in supplemental Table S4 and supplemental Fig. S7.

The HPLC-sub-fractions of the M and L fractions were designated MHP1 to MHP28 and LHP1 to LHP17, respectively (Fig. 2*C* and 2*E*). Based on exploratory experiments using 100 nmol from each of *O*-glycan fractions d to k, which showed that middle fraction 17 was ligand-positive for P[19]VP8* (supplemental Fig. S6), we pooled fractions MHP15 to MHP19 and re-fractionated the pool using a new and better performing HPLC column. Twenty fractions were collected and designated ^M15–19^HP1 to ^M15–19^HP20 (Fig. 2*D*). The 60 fractions thus obtained (MHP1 to MHP14, ^M15–19^HP1 to ^M15–19^HP20, MHP20 to MHP28, and LHP1 to LHP17) were each analyzed by MALDI-MS, quantified and constituted the primary NGL microarray (supplemental Fig. S1 and supplemental Table S4). Five of the fractions were found to be ligand-positive for P[10] and P[19] VP8* proteins. These were ^M15–19^HP13, LHP12, LHP13, LHP14 and LHP17 (Fig. 3 and supplemental Fig. S7).

To isolate and characterize in detail the glycan ligand(s) of the two VP8* proteins we selected one of the more abundant of the four ligand-positive fractions, ^M15–19^HP13 (∼ 8 nmol). Two hexasaccharide-derived NGLs with compositions of dH_1_.H_3_.N_2_-OX and H_2_.N_4_-OY were detected by MS as the main components in this fraction (supplemental Table S4). It should be noted that dH_1_.H_3_.N_2_-OX was also a component detected in the preliminary small scale study in the ligand positive middle fraction 17 (supplemental Fig. S6).

**Fig. 4.**
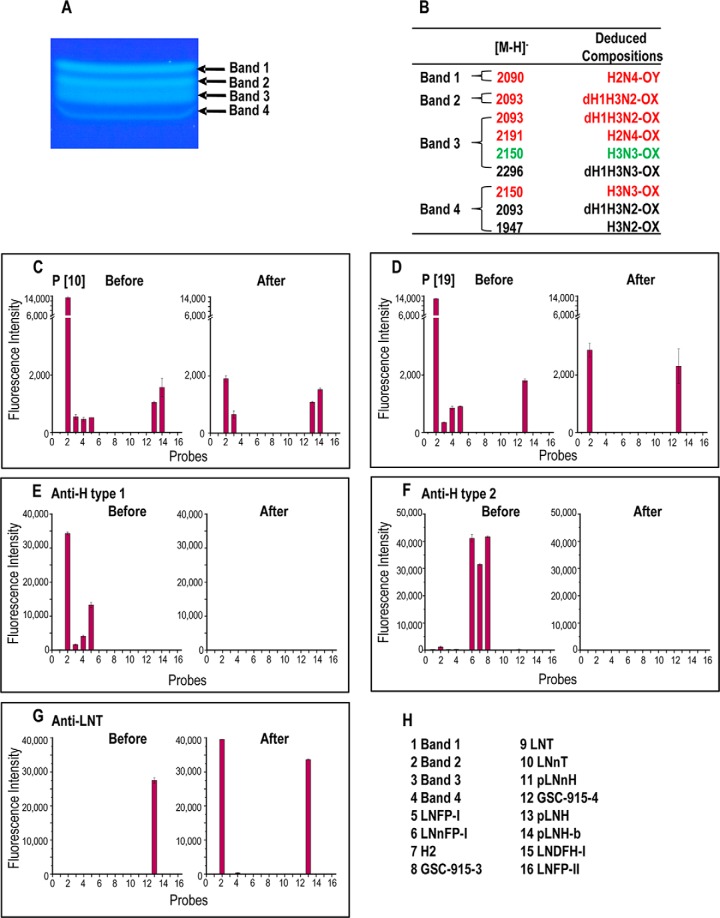
**Fractionation by semi-preparative TLC, MALDI-MS analyses and immuno-sequencing of the components in ligand-positive fraction ^M15–19^HP13.** *A*, High resolution semi-preparative TLC of ^M15–19^HP13; Chromatography was upward; the solvent system was CHCl_3_/MeOH/Water 60:35:8 (by volume). *B*, Molecular ions and deduced monosaccharide compositions of the isolated Bands 1–4, panel (*A*); relative intensities of molecular ions are 80–100 red, 10–70 green, less than 10 black. *C* and *D*, Microarray analyses of the rotavirus P[10] and P[19] VP8* proteins at 50 μg/ml. *E* to *G*, Microarray analyses of antibodies used as immuno-sequencing reagents: anti-H type 1, anti-H type 2 and anti-LNT. *H*, This secondary array (designated “Fucase Array”) included Bands 1–4 and the twelve reference compounds listed in panel *H*; their structures are given in supplemental Table S6. The analyses were performed before and after on-array treatment of the probes with α1–2 fucosidase. The results in *C* to *G*, are the means of fluorescence intensities of duplicate spots printed at 5 fmol/spot, with error bars representing half of the difference between the two values.

A quarter of the fraction ^M15–19^HP13 (∼2 nmol) was sub-fractionated by HPTLC into four components (Fig. 4*A*) designated Bands-1 to -4 with approximate yields, 0.26, 0.26, 1.1, and 0.08 nmol, respectively. We extrapolate that the total yields of the four subfractions were 1.0, 1.0, 4.4 and 0.3 nmol, respectively.

##### A Hexasaccharide Ligand Identified in the Secondary NGL Array

A secondary NGL array was generated containing Bands 1–4, together with 12 reference glycans (Fig. 4*H* and supplemental Table S6). The predominant binding by the VP8* proteins was to Band-2 (Fig 4*C* and 4*D*). From MALDI-MS, Band-2 was assigned as consisting primarily of a single NGL dH_1_.H_3_.N_2_-3OX ([M-H]^−^ at *m*/*z* 2093, Fig. 5*A*), and Band-1, H_2_.N_4_-6OY. Four components were detected in Band-3 and three components in Band-4. An NGL with dH_1_.H_3_.N_2_-3OX composition was among multiple components in Bands-3 and -4 (Fig. 4*B*). Binding analyses with the secondary NGL array showed strongest VP8* binding to Band- 2 (position 2 in Figs. 4*C* and 4*D*); there was only weak binding to Bands 3 and 4 (positions 3 and 4) and no binding to Band 1 (position 1).

**Fig. 5.**
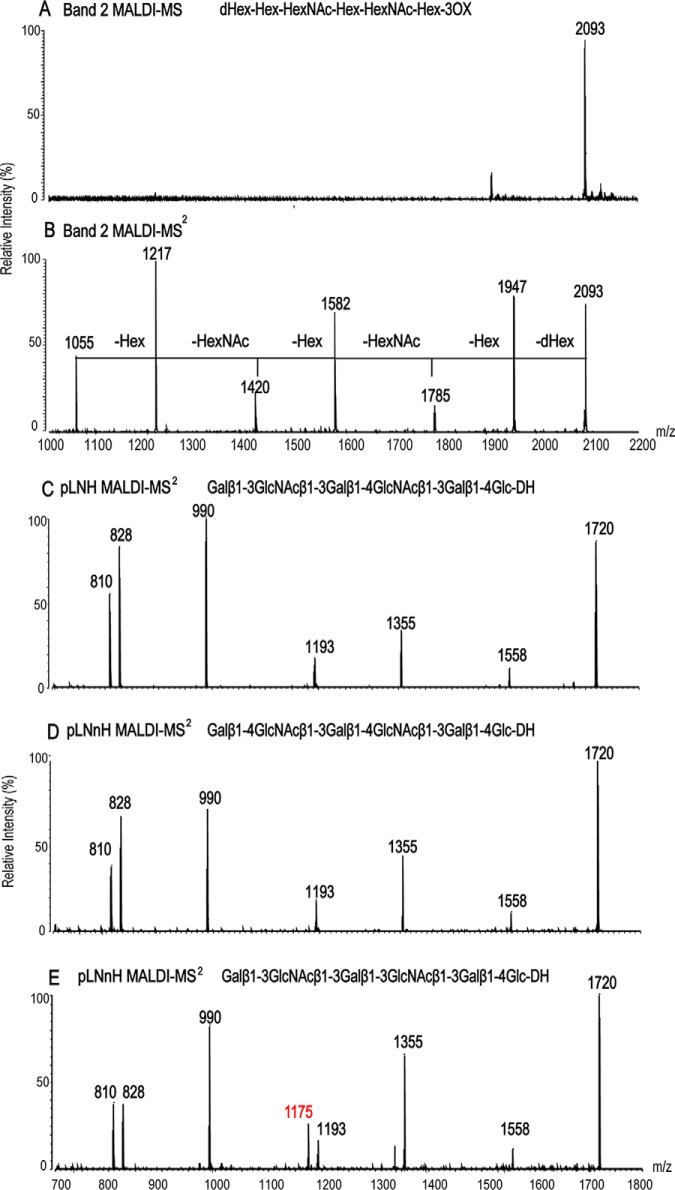
**MALDI-MS analyses of Band 2 and of the NGLs of reference compounds.** *A* and *B*, MALDI-MS and CID-MS/MS of Band 2, respectively. *C* to *E*, MALDI-CID-MS/MS of NGLs of three isomeric hexasaccharides pLNH, pLNnH and pLNH-b. used as reference compounds. The *m*/*z* ion in red in panel *C* is a dehydrated ion characteristic of the “type 1-type 1” backbone sequence in pLNH-b. The corresponding ions are lacking for pLNH and pLNnH that have the “type 1-type 2” sequence, and also for Band 2.

Tandem MS of Band-2 was next performed (Fig. 5*B*). The sequential neutral loss of *m*/*z* 146, 162, 203, 162, 203, and 162 from *m*/*z* 2093 enabled the assignment of Band-2 as having a linear hexasaccharide sequence: dHex–Hex–Hex–NAc–Hex–HexNAc–Hex–3OX, derived from the 3-branch of the core *O*-GalNAcol.

##### On-array Immuno-sequencing and MS of Ligands

“Immuno-sequencing” of Band-2 was then carried out using monoclonal sequence-specific antibodies before and after on-array treatment with α1–2 fucosidase (Fig. 4). Band-2 was strongly bound by mAb 17–206 directed at the blood group H type 1 sequence: Fucα1–2Galβ1–3GlcNAc, but not by mAb BRIC231 directed at blood group H type 2 sequence: Fucα1–2Galβ1–4GlcNAc (Figs. 4*E* and 4*F*). Thus Band-2 was assigned as containing a blood group H type 1 trisaccharide-capping sequence, Fucα1–2Galβ1–3GlcNAc–Hex–HexNAc–Hex–3OX.

Microarray analyses after on-array treatment with α1–2 fucosidase showed loss of binding of Band-2 by both viral proteins as well as anti-H type 1(Fig. 4*C* to 4*E*) and gain of binding by the anti-lacto-*N*-tetraose (LNT) (Fig. 4*G*). This enabled tentative assignment of a tandem “type 1-type 2” backbone sequence as below. Galβ1–3GlcNAcβ1–3Galβ1–4GlcNAcβ1–Gal–3OX.

To our knowledge, the stringency of the anti-LNT for the type 1-type 2 backbone sequence above has not been established. To investigate this, we capitalized on the availability of the three reference compounds (Fig. 4*H*). These were pLNnH (position 11) with the “type 2-type 2” backbone sequence, pLNH (position 13) with the type 1-type 2 backbone sequence, and the novel compound pLNH-b (position 14) isolated in our laboratory (26). This isomer of pLNH has the “type 1-type 1” backbone sequence: Galβ1–3GlcNAcβ1–3Galβ1–3GlcNAcβ1–3Galβ1–4Glc.

Among these three compounds, only pLNH was bound by the anti-LNT mAb (Fig. 4*G*), establishing the stringency of this antibody for the internal type 2 sequence in addition to the stated requirement for the outer type 1 (30). Thus the presence of type 1-type 2 backbone sequence in Band-2 could be unambiguously assigned.

However, the binding of one of the P[10] VP8*, to pLNH-b sequence raised the possibility that Band 2 may contain a second component with the type 1-type-1 backbone sequence. An antibody specific for the pLNH-b sequence is not available to our knowledge. This question could however be addressed by MALDI-CID-MS/MS analyses. For assignment of linkage types in the backbone of Band 2, NGLs of three isomeric hexasaccaharides were analyzed as reference compounds to establish fragmentation patterns. These were: pLNnH with the type 2-type 2 backbone sequence, pLNH with the type 1-type 2”, and pLNH-b with the type 1-type 1 (Fig. 5*C*–5*E*). The three NGLs had identical molecular ions, [M-H]^−^ at *m*/*z* 1720 and all three gave sequence ions at *m*/*z* 1558, 1355, 1193, 990, and 828, with sequential neutral loss of 162, 203, 162, 203, 162, and 162, respectively (in accordance with the linear sequence Gal–GlcNAc–Gal–GlcNAc–Gal–Glc). Only pLNH-b with “type 1-type 1” sequence gave a dehydrated ion (-h) at *m*/*z* 1175 (Fig. 5*E*). This dehydrated ion was more intense than its parent *m*/*z* 1193, and was totally lacking in pLNH and pLNnH with the type 2 internal linkage. This relatively intense dehydrated ion can serve as a marker for the internal type 1 linkage and be used as evidence for there being a pLNH-b type 1-type 1 linkage (26). The lack of the corresponding dehydrated ion at *m*/*z* 1402 arising from *m*/*z* 1420 (Fig. 5*B*) provided strong evidence for the lack of a second component with a type 1-type 1 backbone sequence in Band-2. Therefore, the backbone sequence of Band-2 was assigned as Galβ1–3GlcNAcβ1–3Galβ1–4GlcNAcβ1–Galβ1–3OX.

Collectively the data enable the ligand-positive Band-2 to be assigned as a hexasaccharide with the type 1-terminating blood group H and an internal type 2 sequence, having been derived from a 3-linked branch at core GalNAcol: Fucα1–2Galβ1–3GlcNAcβ1–3Galβ1–4GlcNAcβ1–Galβ–3OX.

With both VP8* proteins, the binding signal given by this hexasaccharide was substantially higher than that given by the pentasaccharide LNFP-I (at position 5, Fig 4*C* and 4*D*) implicating the involvement of the inner 4GlcNAcβ1–Gal–sequence in the ligand for the two VP8* proteins.

The four additional NGL fractions LHP12, LHP13, LHP14, and LHP17 containing larger glycans (8–11mers) that gave binding signals with the two VP8* proteins (Fig. 3) were not investigated in detail; suffice it to say that there was immunochemical evidence for the presence of blood group H type 1 in all four fractions and blood group A type 1 in fractions LHP13, LHP14, and LHP17 (supplemental Table S5 and supplemental Fig. S7). Their monosaccharide compositions deduced by MALDI-MS (supplemental Table S4) are consistent with the immunochemical data. We comment further on these below, in the context of the binding data with a sequence-defined array.

##### Validation of the Beam Search Approach with a Microarray of Sequence-defined Glycan Probes

To validate the assignment of the ligand for the two VP8* proteins identified within PSM neutral *O*-glycome using the beam search arrays, we performed microarray analyses using an array of sequence-defined glycan probes (Table I and supplemental Table S7). In complete accord with beam search array data, both P[10] and P[19] VP8* proteins gave binding signals with the type 1 blood group H sequence, LNFP-I (position 40). Two branched (bivalent) forms, DFiLNO and TFiLNO (1–2, 2, 3) included at positions 38 and 39, respectively, gave stronger binding signals than LNFP I. The binding observed additionally to the type 1 blood group A and B sequences in the sequence-defined array (Table I) is in accord with earlier data (25) reported while this work was underway. These data also support our conclusions that the type 1 blood group A antigen activities we detected in fractions LHP12, LHP13, LHP14, and LHP17 in the beam search array (Fig. 3 and supplemental Table S5) indeed arise from ligand-positive components in PSM *O*-glycome.

**Table I:**
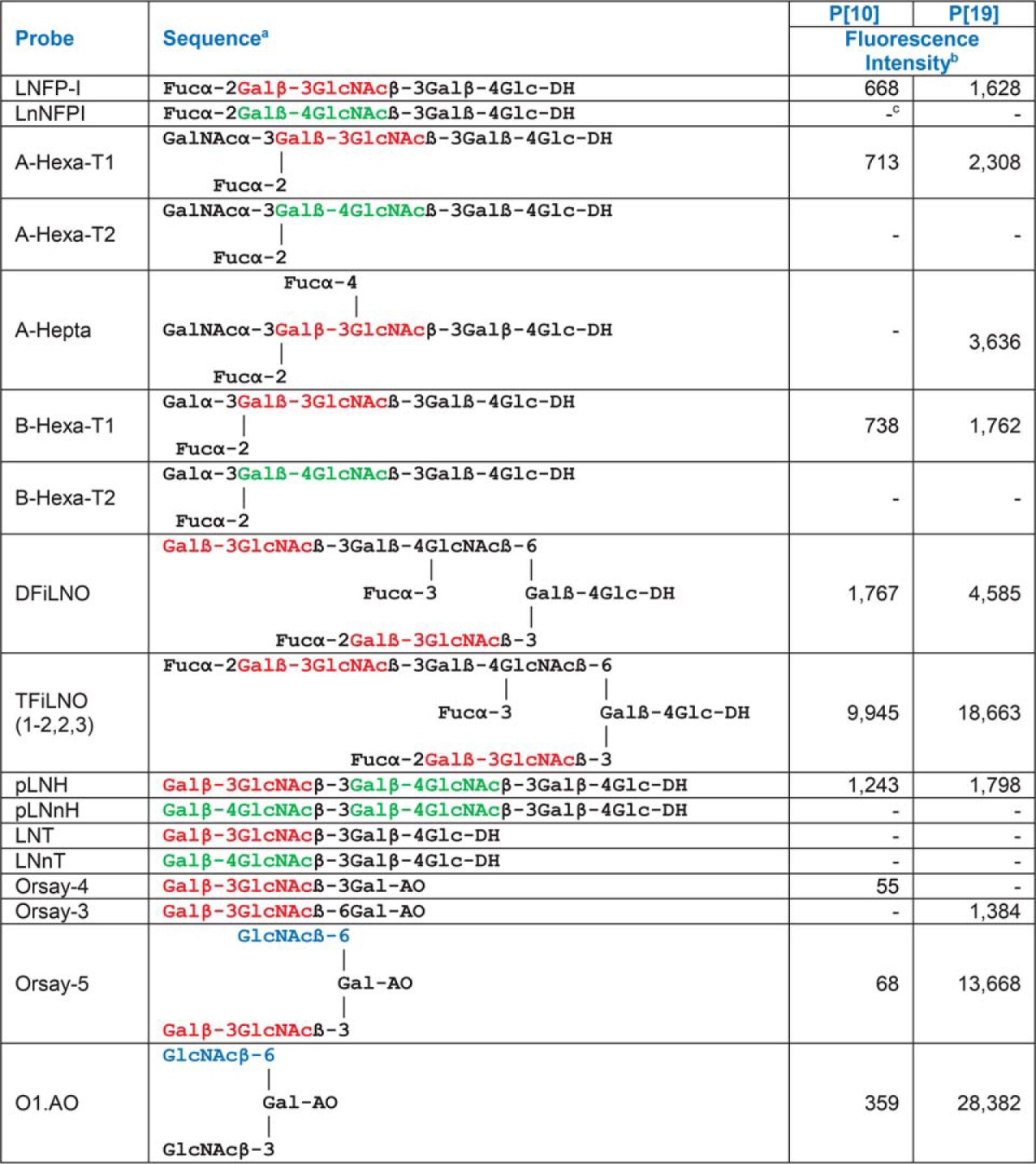
Fluorescence intensities in microarray analyses of the VP8* proteins of P[10] and P[19] (analyzed at 50 μg/ml) to probes selected for comparison in the F77/Ii array. Sequences with type 1 (Galβ1–3GlcNAc) and type 2 (Galβ1–4GlcNAc) terminating backbones, and terminal GlcNAc are shown in red, green, and blue font, respectively

^a^ DH-NGLs were prepared from reducing oligosaccharides by reductive amination with the amino lipid, 1,2-dihexadecyl-sn-glycero-3-phosphoethanolamine (DHPE); AO-NGLs were prepared from reducing oligosaccharides by oxime ligation with an aminooxy (AO) functionalized DHPE (40).

^b^ Fluorescence intensities with probes printed at 5 fmol per spot.

^c^ − Indicates fluorescence intensity less than 1.

##### Additional Specificities Revealed Using the Sequence-defined Screening Array

The analyses using the sequence-defined glycan arrays revealed additional information on glycan sequences bound by the VP8* proteins. (Table I and supplemental Table S7). These are glycan sequences not represented in PSM.

The first was the binding by both proteins to the non-fucosylated type 1-terminating (type 1-type 2) backbone sequence, pLNH (position 10), but not to the type 2-terminating (type 2-type2) analogue pLNnH (position 11). This observation is entirely consistent with the VP8* protein binding observed after on-array fucosidase treatment of Band 2 (Fig. 4*C* and 4*D*). Binding of P[10] VP8* to the pLNH-b, type 1-type-1 sequence was also shown (Fig. 4*C*).

Second, the ALe^b^ sequence (position 46) was bound by the P[19] VP8* but not that of the P[10]. Indeed among the mucins investigated, Cys 733, which was bound by the P[19] and not by the P[10] VP8* protein strongly expresses ALe^b^ (supplemental Fig. S2 and supplemental Table S1). We conclude that Le^b^ sequence may be an important aspect of the difference between the two viruses. This is consistent with our earlier observations (25).

Third, there was pronounced binding by the P[19] but not the P[10] to short branched backbone sequences with a GlcNAcβ1–6-linked to Gal (positions 13 and 14). Binding was considerably weaker to the unbranched GlcNAcβ1–6-linked to Gal capped with Galβ1–3 (position 3).

Fourth was a finding of considerable interest unrelated to the blood group system, namely the binding of the P[19] but not the P[10] VP8* to fungal cell wall type gluco-oligosaccharides (supplemental Table S8). These were of the curdlan and pustulan series (β1–3-linked and β1–6-linked sequences (positions 3–5 and 6–8, respectively) but not cellulose type: β1–4-linked (position 9)).

## DISCUSSION

We have adopted the term “beam search” from computer science where this terminology is used as a process or set of rules to be followed in calculations or problem-solving operations (31). The main features are ability to (a) find the answer or solution efficiently with limited memory requirements; (b) expand possible solutions within the beam width and search solutions or answers within the most promising data set and sub-data set; and (c) combine with other search methods for the ultimate answer (32). Our reasoning to designate the approach described here as beam search is that we aim to search as directly as possible for a ligand or ligands for a recognition protein using a population of natural *O*-glycans that are released from a mucin and may be available in limited amounts. To achieve this, we focus the search on the most promising fractions and sub-fractions. This has the advantage over the TLC plate binding approach (22) as the first line of analysis of the released *O*-glycans. It is of a substantially smaller scale and of a higher throughput as discussed below; and it allows binding analyses concomitant with MS monitoring of glycan content of the fractions during sequential chromatographies.

In the beam search approach, the macromolecule array analysis enables the selection of a ligand-positive, biologically relevant macromolecule (glycoprotein or polysaccharide) for ligand discovery. The primary NGL array is of fractions of released glycans and serves to identify ligand-positive glycan fractions while monitoring glycan compositions by MS. Iterative secondary, and if necessary subsequent arrays, are generated during successive purification steps. Microscale analysis methods are applied including MALDI-MS/MS and immuno-sequencing before and after glycosidase treatments. In this prototype study, the selection of a mucin (PSM) from a macromolecule array, followed by just two glycan arrays enabled the identification and isolation of a ligand-positive hexasaccharide component recognized by the two viral proteins; these were an array of *O*-glycome NGL fractions resolved by HPLC followed by an array containing sub-fractions of a ligand-containing fraction resolved by HPTLC.

There are similarities in the aims of the beam search approach and that of the “shotgun” glycomics of Cummings and colleagues (33–36). Both are to characterize ligands among glycans from natural sources, using sequence analyses by MS/MS and applying sequence-specific lectins and antibodies. There are differences, however, in the design and application of the two approaches. The shotgun glycomics is to present the total glycome from a natural source so that it can be screened to identify physiologically or biologically relevant glycans. The dissection of the glycome is by multidimensional HPLC fractionation aiming to separate individual glycans “as homogeneous as possible” in the first place for printing. Biological questions and applications often come at a later stage. In the beam search array approach, on the other hand, a ligand-bearing macromolecule for a specific recognition system is identified, from which to release, pinpoint and isolate the natural glycan ligand(s); the process involves a ligand-guided iterative fractionation and arraying/probing process focusing on the ligand-positive components which are also the most biologically relevant in the system and context investigated. Hence the generation of a primary and a secondary glycan array from that glycome to cone down on a component expressing the ligand.

The ligand-positive component Band-2, ∼1 nmol of NGL that we isolated from PSM (containing ∼1 μg carbohydrate), amounted to ∼0.2% of the 600 nmol NGLs obtained from 10 mg of *O*-glycan alditols. This was sufficient for unambiguous structural characterization and incorporation into microarrays for up to 8000 binding analyses in our current microarray format. With the chromatogram-binding system, which requires a minimum of 100 pmol of NGLs per lane, only 10 binding analyses can be performed. Thus, the beam search approach is orders of magnitude more sensitive as well as being of higher throughput. The microarray data in the small-scale experiments, using as little as ∼1 mg of *O*-glycan alditols, showed that ligand-containing fractions can be readily detected by this approach with associated MALDI-MS monitoring. The ligand-positive hexasaccharide with the blood group H type 1 sequence (rather than H type 2) has not been described in PSM to our knowledge (37). With optimization of the *O*-glycan derivatization step there is scope for a substantial increase of yields.

The beam search approach calls for sequential fractionation and pooling steps (supplemental Fig. S1). The *O*-glycans released are first size-fractionated prior to oxidative cleavage and lipid conjugation, so that glycans of differing sizes can be separately converted to NGLs, using our established micro-scale conjugation protocols (17, 18), considering average molecular weights of the glycans in the fractions. This also facilitates the MS survey of the variously sized glycans released. After visualizing the resulting NGLs by high performance TLC they are pooled ahead of various chromatographies. The rationale for pooling the NGL population at this stage is to have in one-pot the 3OX- and 6OY-NGLs of similar sizes that arise following the oxidative cleavage of the core GalNAcol of the original branched *O*-glycans of differing sizes. There follows block fractionation of the NGL population using silica gel cartridge before separations by HPLC, and robotic arraying to generate the primary arrays for analyses with carbohydrate-binding proteins of interest. Additional fractionation methods, are used at this stage as appropriate. While considering the advantages of the simplification of glycan profile following the core GalNAcol splitting after the mild periodate oxidation, mention is required that this is at the expense of much of the natural *O*-glycan core which is largely sacrificed. Work is under way to develop further and broaden the beam search technology to encompass core-intact *O*-glycans released by a recently described method (38). This will serve to identify core-specific recognition which may be missed following the core-split. The core-intact, branched *O*-glycans will however have their native heterogeneity and require rigorous fractionations.

The new results from the beam search approach applied to PSM, namely the assignment of the blood group H type 1 sequence as well as the non-fucosylated type 1 backbone sequence as ligands for the two VP8* proteins of the P[10] and P[19] viruses are fully validated by microarray analyses with the sequence-defined glycans. Moreover, the comparative data showed preferential binding of the VP8* proteins to the *O*-glycan component isolated (Band-2) that has a pentasaccharide backbone relative to LNFP I with a tetrasaccharide backbone. Also shown was the ability of the two VP8* proteins to accommodate the blood group A and B determinants (GalNAcα1–3 and Galα1–3, respectively). These findings now account for the lack of a correlation between secretor status and the binding of the two viral proteins to salivary mucins in which the expression of the blood group antigens is controlled by the secretor gene-associated blood group H enzyme (25) (supplemental Table S1).

The additional ligand sequences revealed for the two VP8* proteins by the analyses with the sequence-defined screening arrays are of considerable interest. The three sequences identified that were bound by the P[19] VP8* protein but not by that of the P[10] could potentially come to play in biological contexts other than the pig gastrointestinal tract: The first is the ALe^b^ sequence which is prominent in one of the human epithelial mucins (cyst 733) (supplemental Fig. S2) and would be predicted to occur in the gastrointestinal intestinal epithelial glycoproteins of humans who are blood A Lewis-positive secretors4. The second sequence preferentially bound by the P[19] VP8* protein is GlcNAcβ1–6Gal; this is a candidate terminal motif on poly-N-acetyllactosamine chains of *O*- and *N*-glycans as well as glycolipids unrelated to blood group determinants and secretor status. It will be interesting to investigate the distribution of this motif in tissues as well as possible cross-reactions with the mucin core 2, 4 and 6 sequences that were shown earlier to be bound by the P[19] VP8* protein (25). The third P[19]-associated specificity is non-mammalian; it is toward the fungal polysaccharide-type β1–3 and β1–6 linked glucose sequences. This finding raises the intriguing possibility of interactions of the virus with fungi in the intestinal microbiome where involvement in regulatory processes termed “transkingdom” interactions of which there is an increasing awareness (39). These findings show the important complementarity of the increasing repertoire of sequence-defined glycan arrays with the arrays generated *de novo* from natural sources.

Our immuno-sequencing data highlight the scope for expanding the antibody-tools concomitantly with analyses using sequence-defined glycan arrays to provide reference compounds not only to determine specificities of binding, but also to reveal unsuspected recognition structures that are represented in different physiological contexts.

In conclusion, the beam search approach described here paves the way to *O*-glycome recognition studies in a wide range of basic and medical settings to give new insights into glycan recognition structures in natural microenvironments. A macromolecule array followed by a primary NGL array of the derived, fractionated *O*-glycome NGLs can indeed serve as the starting point for beam search of thousands of diverse binding systems that recognize a given mucin macromolecule.

## DATA AVAILABILITY

The authors declare that the data supporting the findings of this study are compliant with MIRAGE (MIRAGE Glycan Microarray Guidelines - Beilstein-Institut) and are available within the paper and its supplementary information files and Appendix.

## Supplementary Material

Supplemental Data

## Footnotes

^1^ The abbreviations used are:

NGL: neoglycolipid
ADHP: N-aminoacetyl-N-(9-anthracenylmethyl)-1,2-dihexadecyl-sn-glycero-3-phosphoethanolamine
BSM: bovine submaxillary mucin
DHPE: 1,2-dihexadecyl-sn-glycero-3-phosphoethanolamine
ESI-MS: electrospray-ionization induced MS
ESI-CID-MS/MS: electro spray ionization collision induced MS/MS
GST: glutathione S-transferase
HPTLC: high performance thin layer chromatography
LNFPI: lacto-N-fucopentaose I
LNnFPI: lacto-N-neofucopentaose I
LNDFH-I: Lacto-N-difucohexaose
LNFPII: Lacto-N-fucopentaose II
MS: mass spectrometry
MALDI-MS: matrix assisted laser desorption/ionization
PSM: porcine stomach mucin
pLNH: p-Lacto-N-hexaose
pLNnH: p-Lacto-N–neohexaose
UEA-I: Ulex europaeus agglutinin-I.

## References

[1] Andrianifahanana M., Moniaux N., and Batra S. K. (2006) Regulation of mucin expression: Mechanistic aspects and implications for cancer and inflammatory diseases. BBA-Rev. Cancer 1765, 189–222 10.1016/j.bbcan.2006.01.002 16487661

[2] Tran D. T., and Ten Hagen K. G. (2013) Mucin-type O-Glycosylation during Development. J. Biol. Chem. 288, 6921–6929 23329828 10.1074/jbc.R112.418558 PMC3591602

[3] Arike L., and Hansson G. C. (2016) The Densely O-Glycosylated MUC2 Mucin Protects the Intestine and Provides Food for the Commensal Bacteria. J. Mol. Biol. 428, 3221–3229 26880333 10.1016/j.jmb.2016.02.010 PMC4982847

[4] van Kooyk Y., and Rabinovich G. A. (2008) Protein-glycan interactions in the control of innate and adaptive immune responses. Nat. Immunol. 9, 593–601 18490910 10.1038/ni.f.203

[5] Robbe C., Capon C., Coddeville B., and Michalski J. C. (2004) Structural diversity and specific distribution of O-glycans in normal human mucins along the intestinal tract. Biochem. J. 384, 307–316 15361072 10.1042/BJ20040605 PMC1134114

[6] Pinho S. S., and Reis C. A. (2015) Glycosylation in cancer: mechanisms and clinical implications. Nat. Rev. Cancer 15, 540–555 26289314 10.1038/nrc3982

[7] Cummings R. D., and McEver R. P. (2009) C-type Lectins. In: Varki A., Cummings R. D., Esko J. D., Freeze H. H., Stanley P., Bertozzi C. R., Hart G. W., and Etzler M. E., eds. Essentials of Glycobiology 2nd Ed., Cold Spring Harbor Laboratory Press, Cold Spring Harbor, NY 20301239

[8] Rossez Y., Maes E., Darroman T. L., Gosset P., Ecobichon C., Curt M. J. C., Boneca I. G., Michalski J. C., and Robbe-Masselot C. (2012) Almost all human gastric mucin O-glycans harbor blood group A, B or H antigens and are potential binding sites for Helicobacter pylori. Glycobiology 22, 1193–1206 22522599 10.1093/glycob/cws072

[9] Revoredo L., Wang S. J., Bennett E. P., Clausen H., Moremen K. W., Jarvis D. L., Ten Hagen K. G., Tabak L. A., and Gerken T. A. (2016) Mucin-type O-glycosylation is controlled by short- and long-range glycopeptide substrate recognition that varies among members of the polypeptide GalNAc transferase family. Glycobiology 26, 360–376 26610890 10.1093/glycob/cwv108 PMC4767052

[10] Kiessling L. L., and Splain R. A. (2010) Chemical approaches to glycobiology. Annu. Rev. Biochem. 79, 619–653 20380561 10.1146/annurev.biochem.77.070606.100917

[11] Krasnova L., and Wong C. H. (2016) Understanding the Chemistry and Biology of Glycosylation with Glycan Synthesis. Annu. Rev. Biochem. 85, 599–630 27145845 10.1146/annurev-biochem-060614-034420

[12] Watkins W. M. (1980) Biochemistry and Genetics of the ABO, Lewis, and P blood group systems. Adv. Hum. Genet. 10, 1–136, 379–385 6156588 10.1007/978-1-4615-8288-5_1

[13] Kabat E. A. (1982) Philip Levine Award Lecture. Contributions of quantitative immunochemistry to knowledge of blood group A, B, H, Le, I and i antigens. Am. J. Clin. Pathol. 78, 281–292 6180627 10.1093/ajcp/78.3.281

[14] Tang P. W., Gool H. C., Hardy M., Lee Y. C., and Feizi T. (1985) Novel approach to the study of the antigenicities and receptor functions of carbohydrate chains of glycoproteins. Biochem. Biophys. Res. Commun. 132, 474–480 2415127 10.1016/0006-291x(85)91158-1

[15] Yuen C. T., Lawson A. M., Chai W. G., Larkin M., Stoll M. S., Stuart A. C., Sullivan F. X., Ahern T. J., and Feizi T. (1992) Novel sulfated ligands for the cell-adhesion molecule E-selectin revealed by the neoglycolipid technology among O-linked oligosaccharides on an ovarian cystadenoma glycoprotein. Biochemistry 31, 9126–9131 1382586 10.1021/bi00153a003

[16] Yuen C. T., Chai W. G., Loveless R. W., Lawson A. M., Margolis R. U., and Feizi T. (1997) Brain contains HNK-1 immunoreactive O-glycans of the sulfoglucuronyl lactosamine series that terminate in 2-linked or 2,6-linked hexose (mannose). J. Biol. Chem. 272, 8924–8931 9083013 10.1074/jbc.272.14.8924

[17] Stoll M. S., Feizi T., Loveless R. W., Chai W., Lawson A. M., and Yuen C. T. (2000) Fluorescent neoglycolipids. Improved probes for oligosaccharide ligand discovery. Eur. J. Biochem. 267, 1795–1804 10712612 10.1046/j.1432-1327.2000.01178.x

[18] Chai W., Stoll M. S., Galustian C., Lawson A. M., and Feizi T. (2003) Neoglycolipid technology: deciphering information content of glycome. Methods Enzymol. 362, 160–195 12968363 10.1016/S0076-6879(03)01012-7

[19] Fukui S., Feizi T., Galustian C., Lawson A. M., and Chai W. (2002) Oligosaccharide microarrays for high-throughput detection and specificity assignments of carbohydrate-protein interactions. Nat. Biotechnol. 20, 1011–1017 12219077 10.1038/nbt735

[20] Liu Y., Childs R. A., Palma A. S., Campanero-Rhodes M. A., Stoll M. S., Chai W., and Feizi T. (2012) Neoglycolipid-based oligosaccharide microarray system: preparation of NGLs and their noncovalent immobilization on nitrocellulose-coated glass slides for microarray analyses. Methods Mol. Biol. 808, 117–136 22057521 10.1007/978-1-61779-373-8_8

[21] Palma A. S., Feizi T., Childs R. A., Chai W. G., and Liu Y. (2014) The neoglycolipid (NGL)-based oligosaccharide microarray system poised to decipher the meta-glycome. Curr. Opin. in Chem. Biol. 18, 87–94 24508828 10.1016/j.cbpa.2014.01.007 PMC4105633

[22] Gao C., Liu Y., Zhang H. T., Zhang Y. B., Fukuda M. N., Palma A. S., Kozak R. P., Childs R. A., Nonaka M., Li Z., Siegel D. L., Hanfland P., Peehl D. M., Chai W. G., Greene M. I., and Ten F. Z. (2014) Carbohydrate sequence of the prostate cancer-associated antigen F77 assigned by a mucin O-glycome designer array. J. Biol. Chem. 289, 16462–16477 24753245 10.1074/jbc.M114.558932 PMC4047413

[23] Huang P., Xia M., Tan M., Zhong W., Wei C., Wang L., Morrow A., and Jiang X. (2012) Spike protein VP8* of human rotavirus recognizes histo-blood group antigens in a type-specific manner. J. Virol. 86, 4833–4843 22345472 10.1128/JVI.05507-11 PMC3347384

[24] Liu Y., Huang P., Tan M., Liu Y., Biesiada J., Meller J., Castello A. A., Jiang B., and Jiang X. (2012) Rotavirus VP8*: phylogeny, host range, and interaction with histo-blood group antigens. J. Virol. 86, 9899–9910 22761376 10.1128/JVI.00979-12 PMC3446626

[25] Liu Y., Ramelot T. A., Huang P., Liu Y., Li Z., Feizi T., Zhong W., Wu F. T., Tan M., Kennedy M. A., and Jiang X. (2016) Glycan Specificity of P(19) Rotavirus and Comparison with Those of Related P Genotypes. J. Virol. 90, 9983–9996 27558427 10.1128/JVI.01494-16 PMC5068545

[26] Gao C., Zhang Y., Liu Y., Feizi T., and Chai W. (2015) Negative-ion electrospray tandem mass spectrometry and microarray analyses of developmentally regulated antigens based on type 1 and type 2 backbone sequences. Anal. Chem. 87, 11871–11878 26530895 10.1021/acs.analchem.5b03471 PMC4929357

[27] Stoll M. S., and Feizi T. (2009) Software Tools for Storing, Processing and Displaying Carbohydrate Microarray Data After. In: Kettner C., ed. Beilstein Symposium on Glyco-Bioinformatics, pp. 123–140, Beilstein Institute for the Advancement of Chemical Sciences Potsdam, Germany

[28] Liu Y., McBride R., Stoll M., Palma A. S., Silva L., Agravat S., Aoki-Kinoshita K. F., Campbell M. P., Costello C. E., Dell A., Haslam S. M., Karlsson N. G., Khoo K. H., Kolarich D., Novotny M. V., Packer N. H., Ranzinger R., Rapp E., Rudd P. M., Struwe W. B., Tiemeyer M., Wells L., York W. S., Zaia J., Kettner C., Paulson J. C., Feizi T., and Smith D. F. (2016) The minimum information required for a glycomics experiment (MIRAGE) project: improving the standards for reporting glycan microarray-based data. Glycobiology 27, 280–284 10.1093/glycob/cww118 PMC5444268 27993942

[29] Anderson K., Li S. C., and Li Y. T. (2000) Diphenylamine-aniline-phosphoric acid reagent, a versatile spray reagent for revealing glycoconjugates on thin-layer chromatography plates. Anal. Biochem. 287, 337–339 11112283 10.1006/abio.2000.4829

[30] Bry L., Falk P. G., and Gordon J. L. (1996) Genetic engineering of carbohydrate biosynthetic pathways in transgenic mice demonstrates cell cycle-associated regulation of glycoconjugate production in small intestinal epithelial cells. Proc. Natl. Acad. Sci. U.S.A. 93, 1161–1166 8577733 10.1073/pnas.93.3.1161 PMC40049

[31] Ow P. S., and Morton T. E. (1988) Filtered beam search in scheduling. Int. J. Prod. Res. 26, 35–62

[32] Furcy D., and Koenig S. (2005) Limited discrepancy beam search. 19th International Joint Conference on Artificial Intelligence (Ijcai-05), 125–131

[33] Smith D. F., and Cummings R. D. (2014) Investigating virus-glycan interactions using glycan microarrays. Curr. Opin. Virol. 7, 79–87 24995558 10.1016/j.coviro.2014.05.005 PMC4149940

[34] Yu Y., Lasanajak Y., Song X. Z., Hu L. Y., Ramani S., Mickum M. L., Ashline D. J., Prasad B. V. V., Estes M. K., Reinhold V. N., Cummings R. D., and Smith D. F. (2014) Human milk contains novel glycans that are potential decoy receptors for neonatal rotaviruses. Mol. Cell Proteomics 13, 2944–2960 25048705 10.1074/mcp.M114.039875 PMC4223483

[35] Song X., Lasanajak Y., Xia B., Heimburg-Molinaro J., Rhea J. M., Ju H., Zhao C., Molinaro R. J., Cummings R. D., and Smith D. F. (2011) Shotgun glycomics: a microarray strategy for functional glycomics. Nat. Methods 8, 85–90 21131969 10.1038/nmeth.1540 PMC3074519

[36] Byrd-Leotis L., Liu R., Bradley K. C., Lasanajak Y., Cummings S. F., Song X., Heimburg-Molinaro J., Galloway S. E., Culhane M. R., Smith D. F., Steinhauer D. A., and Cummings R. D. (2014) Shotgun glycomics of pig lung identifies natural endogenous receptors for influenza viruses. Proc. Natl. Acad. Sci. U.S.A. 111, E2241–E2250 24843157 10.1073/pnas.1323162111 PMC4050609

[37] Cheng P. F., Snovida S., Ho M. Y., Cheng C. W., Wu A. M., and Khoo K. H. (2013) Increasing the depth of mass spectrometry-based glycomic coverage by additional dimensions of sulfoglycomics and target analysis of permethylated glycans. Anal. Bioanal. Chem. 405, 6683–6695 23797909 10.1007/s00216-013-7128-2

[38] Song X., Ju H., Lasanajak Y., Kudelka M. R., Smith D. F., and Cummings R. D. (2016) Oxidative release of natural glycans for functional glycomics. Nat. Methods 13, 528–534 27135973 10.1038/nmeth.3861 PMC4887297

[39] Pfeiffer J. K. (2016) Host response: Microbiota prime antiviral response. Nat. Microbiol. 1, 15029 27571982 10.1038/nmicrobiol.2015.29

[40] Liu Y., Chai W., Childs R. A., and Feizi T. (2006) Preparation of neoglycolipids with ring-closed cores via chemoselective oxime-ligation for microarray analysis of carbohydrate-protein interactions. Methods Enzymol. 415, 326–340 17116483 10.1016/S0076-6879(06)15020-X

