# Supplementary material for: O-Glycome Beam Search Arrays for Carbohydrate Ligand Discovery
Source: Mol Cell Proteomics. 2017 Nov 28;17(1):121–33. doi: 10.1074/mcp.RA117.000285 (PMC5750842; doi:10.1074/mcp.RA117.000285)
Supplement: Supplemental Data [file supp_17_1_121__index.html]

O-Glycome beam search arrays for carbohydrate ligand discovery — O-glycome beam search arrays — O-Glycome Beam Search Arrays for Carbohydrate Ligand Discovery — O-glycome Beam Search Arrays — Supplemental Data 

# O-Glycome Beam Search Arrays for Carbohydrate Ligand Discovery

## Supplemental Data

- Supplemental data of O-glycome beam search array - Supplementary tables and figures
