# Supplementary material for: O-Glycome Beam Search Arrays for Carbohydrate Ligand Discovery
Source: Mol Cell Proteomics. 2017 Nov 28;17(1):121–33. doi: 10.1074/mcp.RA117.000285 (PMC5750842; doi:10.1074/mcp.RA117.000285)
Supplement: Supplemental Data [file supp_RA117.000285_4909_1_supp_22989_qzcv2y.pdf]

Supplemental Data

O-Glycome beam search array for carbohydrate ligand discovery

Zhen Li[a,1], Chao Gao[a,b,1], Yibing Zhang[a], Angelina S. Palmaa,[c,] Robert A. Childs[a], Lisete M. Silva[a], Yang Liu[d], Xi Jiang[d,e], Yan Liu[a], Wengang Chai[a,2]& Ten Feizi[a,2]

[a] Glycosciences Laboratory, Department of Medicine, Imperial College London,W12 0NN, UK;

[b] Department of Surgery, Beth Israel Deaconess Medical Center, Harvard Medical School, Boston, MA 02215;

[c] Department of Chemistry, UCIBIO-NOVA University of Lisbon, 1099085, Portugal,

[d] Division of Infectious Diseases, Cincinnati Children's Hospital Medical Center and [e] University of Cincinnati College of Medicine, Cincinnati, OH 45229

[1] ZL and CG contributed equally to this work.

[2] To whom correspondence may be addressed E-mail: t.feizi@imperial.ac.uk and w.chai@imperial.ac.uk;

**Table S1**	Microarray analyses of the VP8* proteins of P[10] and P[19] enteroviruses (at 25 µg/mL), antibodies to blood group antigens and the lectin *Ulex europaeus* agglutinin I (UEA-I) using the Macromolecule array of 30 mucin-enriched epithelial glycoproteins.
**Table S2**	Molecular ions and deduced monosaccharide compositions of the major components detected by MALDI-MS analyses and hexose contents in PSM neutral *O*-glycome fractions obtained by Bio-Gel P4 chromatography.
**Table S3**	Molecular ions and deduced compositions from MALDI-MS analyses of the NGLs derived from PSM neutral *O*-glycome fractions.
**Table S4**	Designations, lipid contents, molecular ions and deduced compositions (from MALDI-MS analyses) of PSM neutral *O*-glycome NGL fractions.
**Table S5**	Microarray analyses of the VP8* proteins of enteroviruses P[10] and P[19] (at 50 µg/mL), antibodies to blood group antigens and the lectin *Ulex europaeous* agglutinin I using the PSM neutral *O*-glycome primary beam search array.
**Table S6**	Microarray analyses of the VP8* proteins of the P[10] and P[19] enteroviruses using the secondary beam search array designated 'Fucase Array'.
**Table S7**	Microarray analyses of the enterovirus P[10] and P[19] VP8* proteins (at 50 µg/mL) using the array of sequence-defined probes designated F77/Ii array.
**Table S8**	Microarray analyses on a small glucan array set.
**Scheme S1**	Generation of fluorescent NGLs from a reduced O-glycan by conjugation to the amino lipid N-aminoacetyl-N-(9-anthracenylmethyl)-1,2-dihexadecyl-sn-glycero-3-phosphoethan-olamine (ADHP).
**Fig. S1**	Flow chart of the preparation of *O*-glycome 'beam search' array.
**Fig. S2**	Relative binding intensities of the VP8* proteins of rotavirus P[10] and P[19] (at 25 µg/mL), antibodies and the lectin *Ulex europaeus* agglutinin I in the mucin glycoprotein (primary beam search) array.
**Fig. S3**	Gel filtration chromatography of the products of reductive alkaline hydrolysis from PSM.
**Fig. S4**	High performance TLC of the large scale NGL reaction mixtures of PSM neutral-glycan alditol fractions d to k.
**Fig. S5**	High performance TLC of the pooled PSM neutral *O*-glycome NGL reaction mixture fractionated on a silica cartridge.
**Fig. S6**	Beam search array data from exploratory experiments to explore binding of the VP8* protein of rotavirus P[19] to arrayed neutral *O*-glycome NGL fractions from PSM.
**Fig. S7**	Microarray analysis of VP8* proteins of P[10] and P[19] enteroviruses using PSM neutral *O*-glycome NGL secondary beam search array.
**Appendix**	MIRAGE compliant Glycan Microarray Document

Table S1. Microarray analyses of the VP8* proteins of P[10] and P[19] enteroviruses (at 25 µg/mL), antibodies to blood group antigens and the lectin *Ulex europaeus* agglutinin I (UEA-I) using the macromolecule array of 30 mucin-enriched epithelial glycoproteins.

**Position**[1]	**Mucin**[2]	**Fluorescence signals (150 pg/probe spot)**									
		**P[10]**	**P[19]**	**Anti-H Type 1**	**Anti-H Type 2**	**UEA-I**	**Anti-A**	**Anti-A Type 1**	**Anti-A Type 2**	**Anti-A Le^b**	**Anti-B**
**Enriched cystadenoma mucins**											
1	Cys 350 (Trp) [3]-NS [4]	-[5]	6	-	-	-	261	46	-	2,042	-
2	Cys 444 (Pro) -NS	216	585	-	-	117	1	1,894	3	-	-
3	Cys 444 (Trp) -NS	1276	1967	21	-	2,215	234	298	-	455	-
4	Cys 446 (Pro) -NS	91	134	-	-	-	-	7,952	57	199	-
5	Cys 461 (Pro) -NS	16374	5153	-	-	1,203	-	112	15	-	-
6	Cys 461(Trp) -NS	18393	7586	-	-	6,231	-	28	40	21	-
7	Cys 654 (Pro) -NS	-	-	-	-	-	11	1,776	-	11	-
8	Cys 705 (Pro) -NS	-	-	87	-	-	-	9,350	21	451	-
9	Cys 717 (Pro) -NS	-	47	116	-	-	-	9,225	20	143	-
10	Cys 733 (Pro) -S	-	5936	-	-	5,227	11,626	32,460	32,728	47,640	-
11	Cys 745 (Pro) -NS	712	-	-	-	-	438	1,763	32	63	-
12	Cys 754 (Pro) -NS	-	-	-	-	-	-	1,030	-	8	-
13	Cys 756 (Pro) -NS	82	242	-	-	-	58	3,359	13	146	-
14	Cys 756(Trp) -NS	2756	3102	-	-	-	715	226	9	625	-
15	Cys 762 (Pro) -NS	3498	2109	218	-	-	-	2,207	12	14	-
16	Cys 762(Trp) -NS	9419	5176	2,189	-	49	-	76	-	56	-
17	Cys 765 (Pro) -NS	-	-	12	21	-	-	771	-	13	-
**Meconia**											
18	Meconium B (Pro)-S	18543	6311	13,501	2,625	9,985	36	-	88	-	12,416
19	Meconium Mo (Pro)	5567	3248	-	9	1,405	1,934	-	165	11	-
20	Meconium Wo (Pro)	11469	3932	863	-	1,193	289	-	169	-	-
21	Meconium He (Pro)-S	10014	4913	1,047	-	118	7	-	-	-	11,693
22	Meconium Pa (Pro)-S	9953	4855	8,808	515	5,450	2	-	29	-	7,597
**Purified cystadenoma mucins**											
23	B substance (Pro) -S	3040	4496	926	-	1,387	81	-	9	60	9,261
24	Og 10% 2x (Pep) -NS	13035	4222	-	-	-	2	-	-	-	-
25	Og 10% from 20% (Pep) -NS	13392	6463	-	-	-	-	-	67	-	-

26	N1 20% (Pep) -NS	17072	7781	-	-	3,479	-	2	33	-	-
27	Tij 10% 2x (Pep)-S	9792	5434	1,812	738	7,535	11,972	23,692	18,269	22,773	-
**Other glycoproteins**											
28	HCA	-	-	NI[6]	NI	NI	NI	NI	NI	NI	NI
29	Porcine gastric mucin-'S'	16533	5612	10,256	794	52,423	15,130	26,023	40,912	236	9
30	Bovine submaxillary mucin	3944	2665	-	311	7,168	1,963	6	3	3	-

[1] Position in the array
[2] Samples 1-17 and 23 were mucin enriched preparations from ovarian cystadenoma fluid samples(1); 18-22 were enriched from meconia by protease digestions and ethanol precipitation; Samples 24-27 were purified glycoproteins from ovarian cystadenoma glycoproteins(2); sample 28 was a glycoprotein extract from human lung cancer(3, 4).
[3] Trp, Pro and Pep refer to trypsin, Pronase, or pepsin enzyme treatments for solubilizing mucin-type glycoproteins.
[4]S, NS refer to secretor or non-secretor status where designated in archival records.
[5]-, Fluorescence signals less than 1
[6] NI means not included in the analyses. The P[10] and P[19] VP8* proteins were analysed with Mucin array set 2 and antibodies and UEA-I were analysed on Mucin array set 3.

Table S2. Molecular ions and deduced monosaccharide compositions of the major components detected by MALDI-MS analyses and hexose contents in PSM neutral *O*-glycome fractions obtained by Bio-Gel P4 chromatography.

**Designations**	**[M+Na]$^+$**	**Composition**	**Hexose (mg)**
d[1]	2055[2]	dH1H4N6[3]	18[4]
	2405	dH2H4N7	
	1690	dH1H3N5	
	2932	dH2H6N8	
	2112	H4N7	
e	1325	dH1H2N4	6
	1690	dH1H3N5	
	1179	H2N4	
	1998	dH2H4N5	
	2055	dH1H4N6	
	2112	H4N7	
f	1179	H2N4	4
	1325	dH1H2N4	
	1487	dH1H3N4	
	1471	dH2H2N4	
	1633	dH2H3N4	
	1690	dH1H3N5	
	1893	dH1H3N6	
	1747	H3N6	
	2055	dH1H4N6	
g	1179	H2N4	4
	1122	dH1H2N3	
	1325	dH1H2N4	
	1487	dH1H3N4	
	975	H2N3	
	813	H1N3	

**Designations**	**[M+Na]$^+$**	**Composition**	**Hexose (mg)**
h	1122	dH1H2N3	4
	1179	H2N4	
	1064	dH2H2N2	
	976	H2N3	
	1017	H1N4	
	814	H1N3	
	919	dH1H2N2	
i	814	H1N3	3
	1065	dH2H2N3	
	1122	dH1H2N3	
	919	dH1H2N2	
	976	H2N3	
	1017	H1N4	
	1179	H2N4	
j	611	H1N2	2
	773	H2N2	
	814	H1N3	
	757	dH1H1N2	
	554	dH1H1N1	
	919	dH1H2N2	
	1065	dH2H2N3	
k	611	H1N2	1
	554	dH1H1N1	
			Total 42

[1]Of the thirteen fractions designated a to m, fractions a-c, l and m contained little or no detectable carbohydrate and are not shown.
[2]Relative intensities of molecular ions: 80-100, red; 10-70, green; less than 10, black.
[3]Abbreviations: dH: Deoxyhexose ; H: Hexose; N: *N*-acetylhexosamine or, *N*-acetylhexosaminitol).
[4]Hexose to the nearest milligram.

Table S3. Molecular ions and deduced compositions from MALDI-MS analyses of the NGLs derived from PSM neutral *O*-glycome fractions.

Designation	$[M-H]^-$	Composition	$[M-H]^-$	Composition
d	2353	H3N4-OX[1]	1521	H1N2-OY
	2296	dH1H3N3-OX	1464	dH1H1N1-OY
	1727	dH1H2N1-OX	1668	dH1H1N2-OY
	1931	dH1H2N2-OX	1318	H1N1-OY
	1216	H1-OX	1359	N2-OY
	1784	H2N2-OX	1888	H2N3-OY
	2238	dH2H3N2-OX		
	2645	dH2H3N4-OX		
	1988	H2N3-OX		
	1419	H1N1-OX		
	2092	dH1H3N2-OX		
	2134	dH1H2N3-OX		
	2604	dH2H4N3-OX		
	2662	dH1H4N4-OX		
	2719	H4N5-OX		
	2808	dH2H4N4-OX		
	2865	dH1H4N5-OX		
**e**	2353	H3N4-OX	1521	H1N2-OY
	2296	dH1H3N3-OX	1464	dH1H1N1-OY
	2238	dH2H3N2-OX	1668	dH1H1N2-OY
	1931	dH1H2N2-OX	1318	H1N1-OY
	1784	H2N2-OX	1888	H2N3-OY
	1727	dH1H2N1-OX		
	1565	dH1H1N1-OX		
	2093	dH1H3N2-OX		
	1988	H2N3-OX		
	1216	H1-OX		
	2719	H4N5-OX		
	2500	dH1H3N4-OX		
	2604	dH2H4N3-OX		
	2645	dH2H3N4-OX		
	2661	dH1H4N4-OX		
	2808	dH2H4N4-OX		
**f**	1784	H2N2-OX	1521	H1N2-OY
	1565	dH1H1N1-OX	1668	dH1H1N2-OY
	1931	dH1H2N2-OX	1464	dH1H1N1-OY
	2238	dH2H3N2-OX	1359	N2-OY
	2296	dH1H3N3-OX	1318	H1N1-OY
	2353	H3N4-OX	1156	N1-OY
	2093	dH1H3N2-OX	2135	dH1H2N4-OY
	2500	dH1H3N4-OX	2035	dH1H2N3-OY
	1419	H1N1-OX		
	1216	H1-OX		
**g**	1784	H2N2-OX	1521	H1N2-OY
	1565	dH1H1N1-OX	1668	dH1H1N2-OY
	1932	dH1H2N2-OX	1464	dH1H1N1-OY

	1989	H2N3-OX	1156	N1-OY
	1054	-OX	1318	H1N1-OY
	1216	H1-OX	1359	N2-OY
	1419	H1N1-OX		
	2297	dH1H3N3-OX		
	2354	H3N4-OX		
	2093	dH1H3N2-OX		
	2240	dH2H3N2-OX		
**h**	1419	H1N1-OX	1521	H1N2-OY
	1565	dH1H1N1-OX	1464	dH1H1N1-OY
	1728	dH1H2N1-OX	1668	dH1H1N2-OY
	1786	H2N2-OX	1229	H1N1-OY
	1932	dH1H2N2-OX		
	1216	H1-OX		
**i**	1419	H1N1-OX	1464	dH1H1N1-OY
	1581	H2N1-OX	1521	H1N2-OY
	1728	dH1H2N1-OX	1318	H1N1-OY
	1565	dH1H1N1-OX	1359	N2-OY
	1785	H2N2-OX	1156	N1-OY
	1622	H1N2-OX	1668	dH1H1N2-OY
	1932	dH1H2N2-OX	1831	dH1H2N2-OY
	2240	dH2H3N2-OX		
	2354	H3N4-OX		
	2500	dH1H3N4-OX		
	1216	H1-OX		
**j**	1419	H1N1-OX	1318	H1N1-OY
	1565	dH1H1N1-OX	1464	dH1H1N1-OY
	1581	H2N1-OX	1156	N1-OY
	1728	dH1H2N1-OX	1521	H1N2-OY
	1786	H2N2-OX	1668	dH1H1N2-OY
	1216	H1-OX		
**k**	1419	H1N1-OX	1521	H1N2-OY
	1565	dH1H1N1-OX	1464	dH1H1N1-OY
	1363	dH1H1-OX	1668	dH1H1N2-OY
	1216	H1-OX	1156	N1-OY
	1728	dH1H2N1-OX	1318	H1N1-OY
	1785	H2N2-OX	1888	H2N3-OY
	1932	dH1H2N2-OX		
	2240	dH2H3N2-OX		

[1]OX: -OCH_2-CH(NHAc)-CH_2OH-CH_2-ADHP, derived from a branch 3-linked to core GalNAcol
OY: -OCH_2-CH_2-ADHP derived from a branch 6-linked to core GalNAcol.

Abbreviations for monosaccharides and colour code for relative intensities of molecular ions are as in foot note of **Table S2**.

Table S4. Designations, lipid contents, molecular ions and deduced compositions (from MALDI-MS analyses) of PSM neutral *O*-glycome NGL fractions. These fractions were included in the secondary, *O*-glycome, array; and microarray analysis data of the VP8* proteins of enteroviruses P[10] and P[19] (at 50 µg/mL) are given.

Designations	Amount	$[M-H]^-$	Composition	Position	P[10] Fluorescence signals	P[19]
MHP1[1]	9[2]	?[3]	?[4]	1[5]	-[6]	-
MHP2	3	1522	H1N2-OY	2	-	-
		1465	dH1H1N1-OY			
		1479	?			
		1567	dH1H1N1-OX			
		1623	H1N2-OX			
MHP3	5	1522	H1N2-OY	3	-	-
		1668	dH1H1N2-OY			
		1624	H1N2-OX			
		1728	dH1H2N1-OX			
MHP4	8	1988	H2N3-OX	4	-	-
		2134	dH1H2N3-OX			
		1728	dH1H2N1-OX			
		1623	H1N2-OX			
MHP5	5	1668	dH1H1N2-OY	5	-	-
		1728	dH1H2N1-OX			
MHP6	11	1728	dH1H2N1-OX	6	-	-
		1785	H2N2-OX			
MHP7	24	1785	H2N2-OX	7	-	-
		1728	dH1H2N1-OX			
		1595	?			
MHP8	25	1785	H2N2-OX	8	-	-
		1728	dH1H2N1-OX			
		1931	dH1H2N2-OX			
MHP9	11	1785	H2N2-OX	9	-	-
		1931	dH1H2N2-OX			
		2033	dH1H2N3-OY			
MHP10	33	1931	dH1H2N2-OX	10	-	-
		1785	H2N2-OX			
		1216	H1-OX			
		1830	dH1H2N2-OY			
		1728	dH1H2N1-OX			
MHP11	25	1931	dH1H2N2-OX	11	-	-
		1889	dH1H3N1-OX			
		1728	dH1H2N1-OX			

MHP12	30	1931	dH1H2N2-OX	12	-	-
MHP13	33	1989<br>2135<br>1931	H2N3-OX<br>dH1H2N3-OX<br>dH1H2N2-OX	13	-	-
MHP14	18	1989<br>2135<br>1931<br>2033	H2N3-OX<br>dH1H2N3-OX<br>dH1H2N2-OX<br>dH1H2N3-OY	14	-	-
MHP20	4	2240<br>2151<br>2297	dH2H3N2-OX<br>H3N3-OX<br>dH1H3N3-OX	15	-	-
MHP21	9	2151<br>2240<br>2297<br>2093<br>1931	H3N3-OX<br>dH2H3N2-OX<br>dH1H3N3-OX<br>dH1H3N2-OX<br>dH1H2N2-OX	16	-	-
MHP22	8	2240<br>2297	dH2H3N2-OX<br>dH1H3N3-OX	17	-	-
MHP23	8	2297<br>2354	dH1H3N3-OX<br>H3N4-OX	18	-	-
MHP24	3	2297<br>2354	dH1H3N3-OX<br>H3N4-OX	19	-	-
MHP25	0.8	2354	H3N4-OX	20	-	-
MHP26	0.8	2500<br>2354	dH1H3N4-OX<br>H3N4-OX	21	-	-
MHP27	0.8	2500	dH1H3N4-OX	22	-	-
MHP28	0.8	2646	dH2H3N4-OX	23	-	-
$^{M15\text{-}19}$HP1[7]	1	2090<br>2093	H2N4-OY<br>dH1H3N2-OX	24	-	-
$^{M15\text{-}19}$HP2	1	1420	H1N1-OX	25	-	-
$^{M15\text{-}19}$HP3		1522	H1N2-OY	26	-	-
$^{M15\text{-}19}$HP4	0.8	1582<br>1668	?<br>dH1H1N2-OY	27	-	-
$^{M15\text{-}19}$HP5	1.2	1785<br>1727<br>1931	H2N2-OX<br>dH1H2N1-OX<br>dH1H2N2-OX	28	-	-
$^{M15\text{-}19}$HP6	18	2078	dH2H2N2-OX	29	-	-

		2135	dH1H2N3-OX			
		1931	dH1H2N2-OX			
		1888	H2N3-OY			
M15-19HP7	3.8	2078	dH2H2N2-OX	30	-	-
		1931	dH1H2N2-OX			
M15-19HP8		1989	H2N3-OX	31	-	-
M15-19HP9	2.2	2280	dH2H2N3-OX	32	-	-
M15-19HP10	2.2	2280	dH2H2N3-OX	33	-	-
		2135	dH1H2N3-OX			
M15-19HP11	4	2135	dH1H2N3-OX	34	-	-
		2105	?			
		2093	dH1H3N2-OX			
M15-19HP12	9	1948	H3N2-OX	35	-	-
		2135	dH1H2N3-OX			
		2034	dH1H2N3-OY			
		2093	dH1H3N2-OX			
M15-19HP13	8	2093	dH1H3N2-OX	36	1005	2320
		2090	H2N4-OY			
M15-19HP14	8	2093	dH1H3N2-OX	37	-	-
		1948	H3N2-OX			
		2135	dH1H2N3-OX			
		2034	dH1H2N3-OY			
M15-19HP15	3	2093	dH1H3N2-OX	38	-	-
		2151	H3N3-OX			
M15-19HP16	0.8	2151	H3N3-OX	39	-	-
M15-19HP17	0.8	2240	dH2H3N2-OX	40	-	-
		2297	dH1H3N3-OX			
M15-19HP18	0.8	2354	H3N4-OX	41	-	-
		2297	dH1H3N3-OX			
M15-19HP19	0.8	2297	dH1H3N3-OX	42	-	-
M15-19HP20	0.8	?	?	43	-	-
LHP1	4.5	?	?	44	-	-
LHP2	3	?	?	45	-	-
LHP3	3	?	?	46	-	-
LHP4	1.5	?	?	47	-	-

LHP5	1.5	2093<br>2151<br>2191<br>2296	dH1H3N2-OX<br>H3N3-OX<br>H2N4-OX<br>dH1H3N3-OX	48	-	-
LHP6	1.5	2151<br>2296<br>2337	H3N3-OX<br>dH1H3N3-OX<br>dH1H2N4-OX	49	-	-
LHP7	5	2240	dH2H3N2-OX	50	-	-
LHP8	9	2297	dH1H3N3-OX	51	-	-
LHP9	15	2354	H3N4-OX	52	-	-
LHP10	6	2500<br>2354	dH1H3N4-OX<br>H3N4-OX	53	-	1284
LHP11	8	2500<br>2646	dH1H3N4-OX<br>dH2H3N4-OX	54	-	-
LHP12	9	2646<br>2458	dH2H3N4-OX<br>dH1H4N3-OX	55	897	1746
LHP13	2	2458<br>2662	dH1H4N3-OX<br>dH1H4N4-OX	56	4599	5449
LHP14	3	2605<br>2662	dH2H4N3-OX<br>dH1H4N4-OX	57	3175	5848
LHP15	1	2662<br>2719<br>2751	dH1H4N4-OX<br>H4N5-OX<br>dH3H4N3-OX	58	-	198
LHP 16	2	2719<br>2808<br>2865	H4N5-OX<br>dH2H4N4-OX<br>dH1H4N5-OX	59	-	-
LHP 17	2	2808<br>2970	dH2H4N4-OX<br>dH2H5N4-OX	60	6859	9189
PSM				61	7599	7118

[1] Suffixes M and L refer to HPLC fractions of NGLs with middle and lower migrating features in HPTLC. Other abbreviations and colour codes are as defined in **Tables S2** and S**3.**
[2] The amount of fluorescent lipid in nanomoles.
[3] Fractions MHP1 and LHP1 to LHP4 at the solvent front contained lipid but no detectable carbohydrate.
[4] ? , Unspecified component.
[5] Position in the array.
[6] Fluorescence signals shown are at 5 fmol of lipid per spot; -, signal less than 1.
[7] Subfractions M15 to M19 of the 'middle' migration NGLs were pooled and re-fractionated by HPLC yielding 20 fractions designated $^{M15\text{-}19}$HP1 to $^{M15\text{-}19}$HP20.

Table S5. Microarray analyses of the VP8* proteins of enteroviruses P[10] and P[19] (at 50 µg/mL), antibodies to blood group antigens and the lectin *Ulex europaeous* agglutinin I using the PSM neutral *O*-glycome primary beam search array.

Position	Fractions	Fluoresce signals (5 fmol/probe spot)									
		P[10]	P[19]	Anti-H Type 1	Anti-H Type 2	UEA-I	Anti-A	Anti-A Type 1	Anti-A Type 2	Anti-A Le^b	Anti-B
1	MHP1	- [1]	-	-	-	187	1,448	-	2	-	-
2	MHP2	-	-	-	13,106	4,737	14,463	11	506	-	-
3	MHP3	-	-	-	2,654	1,322	40,299	95	4,655	-	-
4	MHP4	-	-	-	3,009	2,540	29,168	1,232	1,028	-	-
5	MHP5	-	-	-	1,417	629	36,159	166	6,100	-	-
6	MHP6	-	-	2,851	22,983	1,827	15,030	-	224	-	-
7	MHP7	-	-	-	10,154	1,338	9,689	-	51	-	-
8	MHP8	-	-	-	3,198	1,462	16,530	170	85	-	-
9	MHP9	-	-	-	2,310	1,918	42,141	14,104	5,554	-	-
10	MHP10	-	-	-	16,909	1,876	44,433	947	14,640	-	-
11	MHP11	-	-	563	2,630	3,383	23,468	-	1,428	-	-
12	MHP12	-	-	414	1,825	3,048	18,824	-	1,090	-	-
13	MHP13	-	-	-	1,175	4,134	27,147	854	1,016	-	-
14	MHP14	-	-	40	1,113	5,495	24,730	563	2,770	-	-
15	MHP20	-	-	-	793	6,302	31,415	1,552	2,417	-	-
16	MHP21	-	-	-	218	2,761	22,388	2,055	539	-	-
17	MHP22	-	-	66	880	6,489	24,068	-	1,399	-	-
18	MHP23	-	-	149	257	4,068	14,806	-	254	-	-
19	MHP24	-	-	471	13	3,720	21,063	4	759	-	-
20	MHP25	-	-	-	-	1,299	21,285	-	904	-	-
21	MHP26	-	-	90	178	3,562	37,618	92	5,964	-	-
22	MHP27	-	-	915	635	5,418	36,989	216	8,667	-	-
23	MHP28	-	-	689	163	2,414	28,668	-	4,517	-	-
24	$^{M15-19}$ HP1	-	-	-	-	-	-	-	-	-	-
25	$^{M15-19}$ HP2	-	-	-	-	65	-	-	-	-	-
26	$^{M15-19}$ HP3	-	-	-	4,643	2,773	4,744	-	6	-	-
27	$^{M15-19}$ HP4	-	-	-	2,911	1,874	17,288	-	558	-	-
28	$^{M15-19}$ HP5	-	-	-	1,288	2,558	13,051	36	441	-	-
29	$^{M15-19}$ HP6	-	-	-	2,231	6,302	19,396	72	1,189	-	-
30	$^{M15-19}$ HP7	-	-	-	7,418	13,950	13,566	-	475	-	-
31	$^{M15-19}$ HP8	-	-	108	2,813	8,278	31,440	1,268	2,000	-	-
32	$^{M15-19}$ HP9	-	-	-	103	3,197	18,556	-	676	-	-
33	$^{M15-19}$ HP10	-	-	-	934	8,310	38,387	7	8,592	-	-
34	$^{M15-19}$ HP11	-	-	17,155	1,452	5,018	38,770	-	11,195	-	-
35	$^{M15-19}$ HP12	-	-	2,169	5,647	11,169	28,213	-	4,145	-	-
36	$^{M15-19}$ HP13	1,005	2,320	14,767	-	1,468	16,895	1,287	200	-	-
37	$^{M15-19}$ HP14	-	-	825	1,425	6,293	21,266	-	1,069	-	-
38	$^{M15-19}$ HP15	-	-	517	350	5,363	19,195	2,889	78	-	-
39	$^{M15-19}$ HP16	-	-	1,411	240	4,790	27,064	2,277	325	-	-
40	$^{M15-19}$ HP17	-	-	1,429	399	6,086	19,821	477	250	-	-
41	$^{M15-19}$ HP18	-	-	-	-	652	3,882	-	-	-	-
42	$^{M15-19}$ HP19	-	-	38	-	3	13	11	-	-	-

43	M15-19 HP20	-	-	-	-	-	-	-	-	-	-
44	LHP1	-	-	-	-	110	297	-	-	-	-
45	LHP2	-	-	-	-	250	1,875	-	44	-	-
46	LHP3	-	-	-	869	120	580	-	-	-	-
47	LHP4	-	-	-	12	428	2,529	22	-	-	-
48	LHP5	-	-	2,349	732	1,857	17,473	1,260	111	-	-
49	LHP6	-	-	-	-	468	20,522	7,298	133	-	-
50	LHP7	-	-	-	263	3,822	17,090	442	532	-	-
51	LHP8	-	-	1,572	629	2,743	15,258	179	287	-	-
52	LHP9	-	-	1,013	314	2,465	20,428	554	659	-	-
53	LHP10	-	1,284	4,788	1,589	6,200	38,178	3,789	6,229	-	-
54	LHP11	-	-	1,846	687	2,688	36,040	1,623	8,053	-	-
55	LHP12	897	1,746	19,645	4,471	3,374	37,503	1,446	6,532	-	-
56	LHP13	4,599	5,449	19,320	5,434	6,258	34,728	13,216	2,727	-	-
57	LHP14	3,175	5,848	17,783	7,050	5,111	31,231	7,137	2,161	-	-
58	LHP15	-	198	5,194	2,744	3,545	27,602	4,546	1,528	-	-
59	LHP16	-	-	4,468	3,101	5,967	26,629	2,538	1,847	-	-
60	LHP17	6,859	9,189	24,131	9,196	28,062	42,635	16,843	8,397	-	-
61	PSM	7,599	7,118	19,941	10,010	62,424	45,300	18,105	30,694	-	-

[1] Fluorescence signal less than 1.

Table S6. Microarray analyses of the VP8* proteins of the P[10] and P[19] enteroviruses (at 50 µg/mL) using the secondary array designated 'Fucase Array'. Four sub-fractions, Bands 1-4, isolated by semi-preparative TLC from fraction M15-19HP13 and twelve sequence-defined NGL probes constituted the array which was used for analyses before and after on-array treatment with α1-2 fucosidase.

Position	Probes	Composition/Sequence	Fluorescence signals (5 fmol/probe spot)									
			P[10]		P[19]		Anti-H type 1		Anti-H type 2		Anti-LNT	
			Before	After	Before	After	Before	After	Before	After	Before	After
1	Band 1	H2N4-OY	-	-	-	-	162	-	280	-	-	-
2	Band 2	dH1H3N2-OX	13,432	1,901	12,969	2,874	34,298	-	1,213	-	-	39,543
3	Band 3	dH1H3N2-OX<br>H3N3-OX<br>H2N4-OX<br>dH1H3N3-OX	541	648	347	-	1,647	-	260	-	-	-
4	Band 4	H3N2-OX<br>dH1H3N2-OX<br>H3N3-OX	456	-	852	-	4,123	-	304	-	-	309
5	LNFP-I	Fucα-2Galβ-3GlcNAcβ-3Galβ-4Glc-DH[1]	519	-	907	-	13,296	-	35	-	-	-
6	LNnFP-I	Fucα-2Galβ-4GlcNAcβ-3Galβ-4Glc-DH	-	-	-	-	-	-	41,070	28	-	-
7	H2 (with H2+Fuc)*	Fucα-2Galβ-4GlcNAcβ-3Galβ-4GlcNAcβ-3Galβ-4Glcβ-Cer[2]	-	-	-	-	-	-	31,484	37	-	-
8	GSC-915-3	Fucα-2Galβ-4GlcNAcβ-6Galβ-4GlcNAcβ-3Galβ-4Glc-DH	-	-	-	-	-	-	41,601	41	17	-
9	LNT	Galβ-3GlcNAcβ-3Galβ-4Glc-DH	-	-	-	-	-	-	-	45	-	-
10	LNnT	Galβ-4GlcNAcβ-3Galβ-4Glc-DH	-	-	-	-	-	-	-	-	-	-
11	pLNnH	Galβ-4GlcNAcβ-3Galβ-4GlcNAcβ-3Galβ-4Glc-DH	-	-	-	-	-	-	-	-	-	-
12	GSC-915-4	Galβ-4GlcNAcβ-6Galβ-4GlcNAcβ-3Galβ-4Glc-DH	-	-	-	-	-	-	224	-	-	-
13	pLNH	Galβ-3GlcNAcβ-3Galβ-4GlcNAcβ-3Galβ-4Glc-DH	1,046	1,082	1,800	2,310	-	-	-	-	27,501	33,651
14	pLNH-b	Galβ-3GlcNAcβ-3Galβ-3GlcNAcβ-3Galβ-4Glc-DH	1,571	1,525	3	-	-	-	-	-	-	-
15	LNDFH-I	Fucα-2Galβ-3GlcNAcβ-3Galβ-4Glc-DH<br>| Fucα-4	-	-	-	-	-	-	-	-	-	-
16	LNFP-II	Galβ-3GlcNAcβ-3Galβ-4Glc-DH<br>| Fucα-4	-	-	-	-	-	-	-	-	-	-

[1] DH, designates NGLs prepared from reducing oligosaccharides by reductive amination with the amino lipid, 1,2-dihexadecyl-sn-glycero-3-phosphoethanolamine;

[2] Cer, natural glycolipid with various ceramide moieties.

*Asterisk indicates the the presecne of a minor component with an additional fucose residue detected by MALDI-MS; most likely these are part of a difucosylated Le^{y} sequence(5). Other abbreviations are as in **Tables S2** and **S3**.

Table S7. Microarray analyses of the enterovirus P[10] and P[19] VP8* proteins (at 50 µg/mL) using the array of sequence-defined probes designated F77/Ii array .

			**fluorescence signals (5 fmol/probe spot)**	
**Position**	**Probes**	**Structure**	**P[10]**	**P[19]**
1	Orsay-1-AO	Galβ-4GlcNAcβ-6Gal-AO[1]	-[2]	-
2	Orsay-2-AO	Galβ-4GlcNAcβ-3Gal-AO	-	-
3	Orsay-3-AO	Galβ-3GlcNAcβ-6Gal-AO	-	1,384
4	Orsay-4-AO	Galβ-3GlcNAcβ-3Gal-AO	55	-
5	LNT	Galβ-3GlcNAcβ-3Galβ-4Glc-DH[1]	-	-
6	LNnT	Galβ-4GlcNAcβ-3Galβ-4Glc-DH	-	-
7	LSTa	NeuAcα-3Galβ-3GlcNAcβ-3Galβ-4Glc-DH	-	-
8	LSTc	NeuAcα-6Galβ-4GlcNAcβ-3Galβ-4Glc-DH	36	-
9	LSTd	NeuAcα-3Galβ-4GlcNAcβ-3Galβ-4Glc-DH	-	-
10	pLNH	Galβ-3GlcNAcβ-3Galβ-4GlcNAcβ-3Galβ-4Glc-DH	1,243	1,798
11	pLNnH	Galβ-4GlcNAcβ-3Galβ-4GlcNAcβ-3Galβ-4Glc-DH	-	-
12	LNnO	Galβ-4GlcNAcβ-3Galβ-4GlcNAcβ-3Galβ-4GlcNAcβ-3Galβ-4Glc-DH	-	-
13	O1-AO	GlcNAcβ-6<br>| Gal-AO<br>| GlcNAcβ-3	359	28,382
14	Orsay-5-AO	GlcNAcβ-6<br>| Gal-AO<br>| Galβ-3GlcNAcβ-3	68	13,668
15	Orsay-6-AO	Galβ-4GlcNAcβ-6<br>| Gal-AO<br>| Galβ-3GlcNAcβ-3	-	-

16	Orsay-7-AO	Galβ-4GlcNAcβ-6<br>| Gal-AO<br>| Galβ-4GlcNAcβ-3	-	-
17	LNH	Galβ-4GlcNAcβ-6<br>| Galβ-4Glc-DH<br>| Galβ-3GlcNAcβ-3	-	65
18	LNnH	Galβ-4GlcNAcβ-6<br>| Galβ-4Glc-DH<br>| Galβ-4GlcNAcβ-3	-	92
19	MSLNH	NeuAcα-6Galβ-4GlcNAcβ-6<br>| Galβ-4Glc-DH<br>| Galβ-3GlcNAcβ-3	-	-
20	MSLNnH-I	Galβ-4GlcNAcβ-6<br>| Galβ-4Glc-DH<br>| NeuAcα-6Galβ-3GlcNAcβ-3	-	-
21	DSLNnH	NeuAcα-6Galβ-4GlcNAcβ-6<br>| Galβ-4Glc-DH<br>| NeuAcα-6Galβ-4GlcNAcβ-3	-	-
22	iLNO	Galβ-3GlcNAcβ-3Galβ-4GlcNAcβ-6<br>| Galβ-4Glc-DH<br>| Galβ-3GlcNAcβ-3	509	141

23	LND	Galβ-4GlcNAcβ-6<br>Galβ-4GlcNAcβ-6<br>Galβ-3GlcNAcβ-3 Galβ-4Glc-DH<br>Galβ-3GlcNAcβ-3	-	-
24	Nonaosylceramide	GlcNAcβ-6<br>GlcNAcβ-6 Galβ-4GlcNAcβ-3Galβ-4Glcβ-Cer<br>Galβ-4GlcNAcβ-3<br>GlcNAcβ-3	59	205
25	I-octaosylceramide	Galβ-4GlcNAcβ-6<br>Galβ-4GlcNAcβ-3Galβ-4Glcβ-Cer<br>Galβ-4GlcNAcβ-3	-	-
26	I-dodecaosylceramide	Galβ-4GlcNAcβ-6<br>Galβ-4GlcNAcβ-6 Galβ-4GlcNAcβ-3Galβ-4Glcβ-Cer<br>Galβ-4GlcNAcβ-3<br>Galβ-4GlcNAcβ-3	-	-
27	B-like decaosylceramide	Galα-3Galβ-4GlcNAcβ-6<br>Galβ-4GlcNAcβ-3Galβ-4Glcβ-Cer<br>Galα-3Galβ-4GlcNAcβ-3	-	-
28	B-like pentadecaosylceramide	Galα-3Galβ-4GlcNAcβ-6<br>Galα-3Galβ-4GlcNAcβ-6 Galβ-4GlcNAcβ-3Galβ-4Glcβ-Cer<br>Galβ-4GlcNAcβ-3<br>Galα-3Galβ-4GlcNAcβ-3	-	236

<table>
<tr><td>29</td><td>LNFP-II</td><td>Galβ-3GlcNAcβ-3Galβ-4Glc-DH
|
Fucα-4</td><td>-</td><td>-</td></tr>
<tr><td>30</td><td>Leb-hexaosylceramide</td><td>Fucα-2Galβ-3GlcNAcβ-3Galβ-4Glcβ-Cer
|
Fucα-4</td><td>75</td><td>-</td></tr>
<tr><td>31</td><td>LNFP-III</td><td>Galβ-4GlcNAcβ-3Galβ-4Glc-DH
|
Fucα-3</td><td>-</td><td>147</td></tr>
<tr><td>32</td><td>LNnDFH-I</td><td>Fucα-2Galβ-4GlcNAcβ-3Galβ-4Glc-DH
|
Fucα-3</td><td>-</td><td>-</td></tr>
<tr><td>33</td><td>MFLNH-I</td><td>Galβ-4GlcNAcβ-6
|
Galβ-4Glc-DH
|
Fucα-2Galβ-3GlcNAcβ-3</td><td>5</td><td>-</td></tr>
<tr><td>34</td><td>DFLNH(b)</td><td>Galβ-4GlcNAcβ-6
| |
Fucα-3 Galβ-4Glc-DH
|
Galβ-3GlcNAcβ-3
|
Fucα-4</td><td>111</td><td>-</td></tr>
<tr><td>35</td><td>TFLNH</td><td>Galβ-4GlcNAcβ-6
| |
Fucα-3 Galβ-4Glc-DH
|
Fucα-2Galβ-3GlcNAcβ-3
|
Fucα-4</td><td>-</td><td>93</td></tr>
<tr><td>36</td><td>DFLNnH</td><td>Galβ-4GlcNAcβ-6
| |
Fucα-3 Galβ-4Glc-DH
|
Galβ-4GlcNAcβ-3
|
Fucα-3</td><td>-</td><td>-</td></tr>
</table>

37	MSDFLNnH-AO	Fucα-2Galβ-4GlcNAcβ-6<br>Fucα-3 Galβ-4Glc-AO<br>NeuAcα-6Galβ-4GlcNAcβ-3	-	-
38	DFiLNO	Galβ-3GlcNAcβ-3Galβ-4GlcNAcβ-6<br>Fucα-3 Galβ-4Glc-DH<br>Fucα-2Galβ-3GlcNAcβ-3	1,768	4,585
39	TFiLNO(1-2,2,3)	Fucα-2Galβ-3GlcNAcβ-3Galβ-4GlcNAcβ-6<br>Fucα-3 Galβ-4Glc-DH<br>Fucα-2Galβ-3GlcNAcβ-3	9,945	18,663
40	LNFP-I	Fucα-2Galβ-3GlcNAcβ-3Galβ-4Glc-DH	668	1,628
41	LNnFP-I	Fucα-2Galβ-4GlcNAcβ-3Galβ-4Glc-DH	-	-
42	H2 (with H2+Fuc)*	Fucα-2Galβ-4GlcNAcβ-3Galβ-4GlcNAcβ-3Galβ-4Glcβ-Cer	-	-
43	H3 (with H3-Fuc)*	Fucα-2Galβ-4GlcNAcβ-6<br>Galβ-4GlcNAcβ-3Galβ-4Glc-Cer<br>Fucα-2Galβ-4GlcNAcβ-3	-	-
44	A-Hexa-T1	GalNAcα-3Galβ-3GlcNAcβ-3Galβ-4Glc-DH<br>Fucα-2	713	2,308
45	A-Hexa-T2	GalNAcα-3Galβ-4GlcNAcβ-3Galβ-4Glc-DH<br>Fucα-2	-	-
46	A-Hepta	Fucα-4<br>GalNAcα-3Galβ-3GlcNAcβ-3Galβ-4Glc-DH<br>Fucα-2	-	3,636
47	Ab (with Ab+Fuc)*	GalNAcα-3Galβ-4GlcNAcβ-3Galβ-4GlcNAcβ-3Galβ-4Glcβ-Cer<br>Fucα-2	61	-

48	Ad (with Ad+Fuc)*	GalNAcα-3Galβ-4GlcNAcβ-6<br>Fucα-2 Galβ-4GlcNAcβ-3Galβ-4Glcβ-Cer<br>GalNAcα-3Galβ-4GlcNAcβ-3Galβ-4GlcNAcβ-3<br>Fucα-2	-	-
49	B-Hexa-T1	Galα-3Galβ-3GlcNAcβ-3Galβ-4Glc-DH<br>Fucα-2	739	1,762
50	B-Hexa-T2	Galα-3Galβ-4GlcNAcβ-3Galβ-4Glc-DH<br>Fucα-2	-	-
51	B-penta-AO	Galα-3Galβ-4Glc-AO<br>Fucα-2<br>Fucα-3	-	-
52	B-III dodecaosylceramide	Galα-3Galβ-4GlcNAcβ-6<br>Fucα-2 Galβ-4GlcNAcβ-3Galβ-4Glcβ-Cer<br>Galα-3Galβ-4GlcNAcβ-3<br>Fucα-2	273	-
53	B-IV tetradecaosylceramide	Galα-3Galβ-4GlcNAcβ-6<br>Fucα-2 Galβ-4GlcNAcβ-3Galβ-4Glcβ-Cer<br>Galα-3Galβ-4GlcNAcβ-3Galβ-4GlcNAcβ-3<br>Fucα-2	-	-
54	PSM-F1H2HN3-OY	Fucα-2Galβ-4GlcNAcβ-6<br>Galβ-4GlcNAcβ-OY<br>GlcNAcβ-3	-	-

<table>
<tr><td>55</td><td>PIII (proposed)</td><td>Fucα-2Galβ-3GlcNAcβ-6
|
Galβ-4GlcNAcβ-3Galβ-4Glc-Cer
|
Fucα-2Galβ-3GlcNAcβ-3

Fucα-2Galβ-4GlcNAcβ-6
|
Galβ-4GlcNAcβ-3Galβ-4Glc-Cer
|
Galβ-3GlcNAcβ-3

Fucα-2Galβ-4GlcNAcβ-6
| |
Fucα-3 Galβ-4GlcNAcβ-3Galβ-4Glc-Cer
|
Fucα-2Galβ-3GlcNAcβ-3</td><td>-</td><td>-</td></tr>
<tr><td>56</td><td>PIII de Fuc (according to proposed structures)</td><td>Galβ-3GlcNAcβ-6
|
Galβ-4GlcNAcβ-3Galβ-4Glc-Cer
|
Galβ-3GlcNAcβ-3

Galβ-4GlcNAcβ-6
|
Galβ-4GlcNAcβ-3Galβ-4Glc-Cer
|
Galβ-3GlcNAcβ-3

Galβ-4GlcNAcβ-6
| |
Fucα-3 Galβ-4GlcNAcβ-3Galβ-4Glc-Cer
|
Galβ-3GlcNAcβ-3</td><td>-</td><td>100</td></tr>
</table>

57	GSC-915	Fucα-2Galβ-4GlcNAcβ-6<br>| Galβ-4GlcNAcβ-3Galβ-4Glc-DH<br>| Galβ-4GlcNAcβ-3	-	153
58	GSC-915-2	Fucα-2Galβ-4GlcNAcβ-6<br>| Galβ-4GlcNAcβ-3Galβ-4Glc-DH<br>| GlcNAcβ-3	-	-
59	GSC-915-3	Fucα-2Galβ-4GlcNAcβ-6Galβ-4GlcNAcβ-3Galβ-4Glc-DH	-	3
60	GSC-915-4	Galβ-4GlcNAcβ-6Galβ-4GlcNAcβ-3Galβ-4Glc-DH	-	15

[1] The oligosaccharide probes are all lipid-linked, neoglycolipids (NGLs) or glycosylceramides and are from the collection assembled in the course of research in Glycosciences Laboratory. NGLs designated DH are prepared from reducing oligosaccharides by reductive amination with the amino lipid, 1,2-dihexadecyl-sn-glycero-3-phosphoethanolamine (DHPE); AO-NGLs were prepared from reducing oligosaccharides by oxime ligation with an aminooxy (AO) functionalized DHPE(6); Cer, natural glycolipids with various ceramide moieties.

[2] Fluorescence signals less than 1

*Asterisks indicate glycolipid preparations that contain a minor component with an additional fucose residue detected by MALDI-MS; most likely these are part of the difucosylated Le^y sequence (5).

Table S8. Microarray analyses of. P[10] and P[19] VP8* proteins at 100 µg/mL on an array set of 7 gluco-oligosaccharide NGLs and LNFP1 and LNnFP1 NGLs as controls; data are at 5 fmol/spot .

Number	Probe	Sequence[1]	P[10]	P[19]
			Fluorescence Intensity[2]	
1	LNFPI	Fucα-2Galβ-3GlcNAcβ-3Galβ-4Glc-DH	1,053	3,188
2	LNnFPI	Fucα-2Galβ-4GlcNAcβ-3Galβ-4Glc-DH	-[3]	-
3	Lam-4-AO	Glcβ-3Glcβ-3Glcβ-3Glc-AO	-	1,061
4	Lam-6-AO*	Glcβ-3Glcβ-3Glcβ-3Glcβ-3Glcβ-3Glc-AO	-	2,812
5	Curd-8-AO*	Glcβ-3Glcβ-3Glcβ-3Glcβ-3Glcβ-3Glcβ-3Glcβ-3Glc-AO	-	3,397
6	Pust-4-AO	Glcβ-6Glcβ-6Glcβ-6Glc-AO	-	3,781
7	Pust-6-AO	Glcβ-6Glcβ-6Glcβ-6Glcβ-6Glcβ-6Glc-AO	-	2,502
8	Pust-8-AO*	Glcβ-6Glcβ-6Glcβ-6Glcβ-6Glcβ-6Glcβ-6Glcβ-6Glc-AO	-	1,075
9	Cello-6-AO*	Glcβ-4Glcβ-4Glcβ-4Glcβ-4Glcβ-4Glc-AO	-	-

[1] DH-NGLs were prepared from reducing oligosaccharides by reductive amination with the amino lipid, 1,2-dihexadecyl-sn-glycero-3-phosphoethanolamine (DHPE); AO-NGLs were prepared from reducing oligosaccharides by oxime ligation with an aminooxy (AO) functionalized DHPE(6).
[2] Fluorescence intensities with probes printed at 5 fmol per spot.
[3] - Indicates fluorescence intensity less than 1.
*Asterisks indicate the major components when multiple components are present.

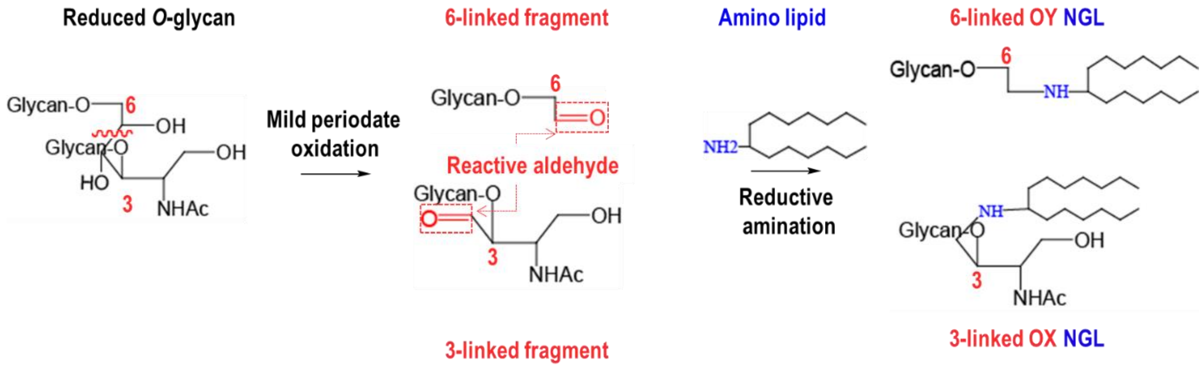

Scheme S1. Generation of fluorescent NGLs from a reduced *O*-glycan by conjugation to the amino lipid N-aminoacetyl-N-(9-anthracenylmethyl)-1,2-dihexadecyl-sn-glycero-3-phosphoethan-olamine (ADHP).

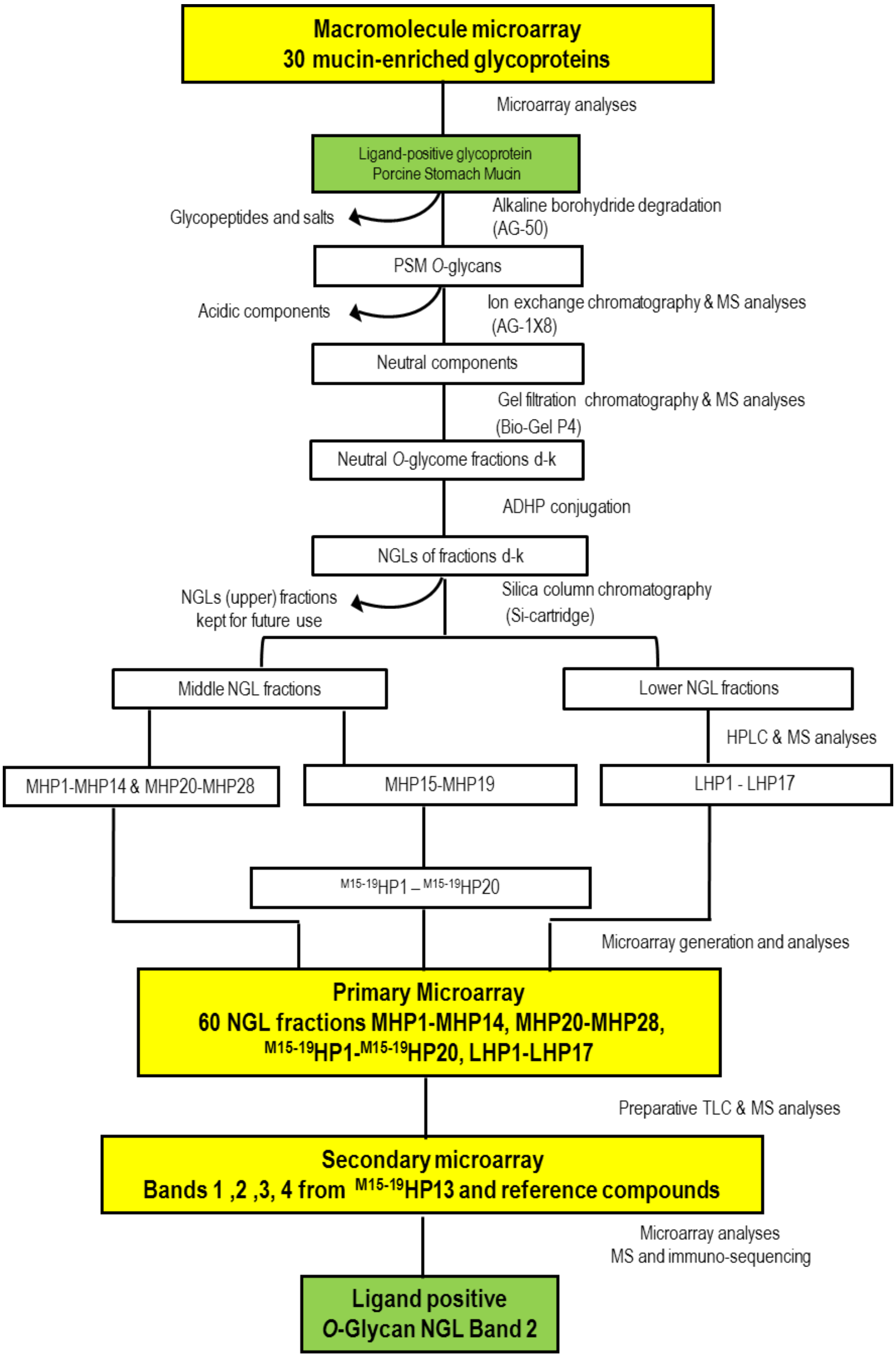

Fig. S1. Flow chart of steps in the *O*-glycome 'beam search' approach highlighting the three microarrays (yellow) that led to the identification of ligand positive components (green), and the pinpointing and elucidation of an *O*-glycan ligand for the VP8* proteins of rotaviruses P[10] and P[19]. Microarray analyses of the VP8* proteins of rotaviruses P[10] and P[19] on a primary microarray consisting of 30 mucin-enriched glycoproteins enabled the selection of porcine stomach mucin (PSM) as the ligand-bearing mucin from which to generate NGLs of the released *O*-glycan alditols for the secondary and tertiary arrays of NGLs. The ligand positive mucin and derived ligand positive NGL are in green.

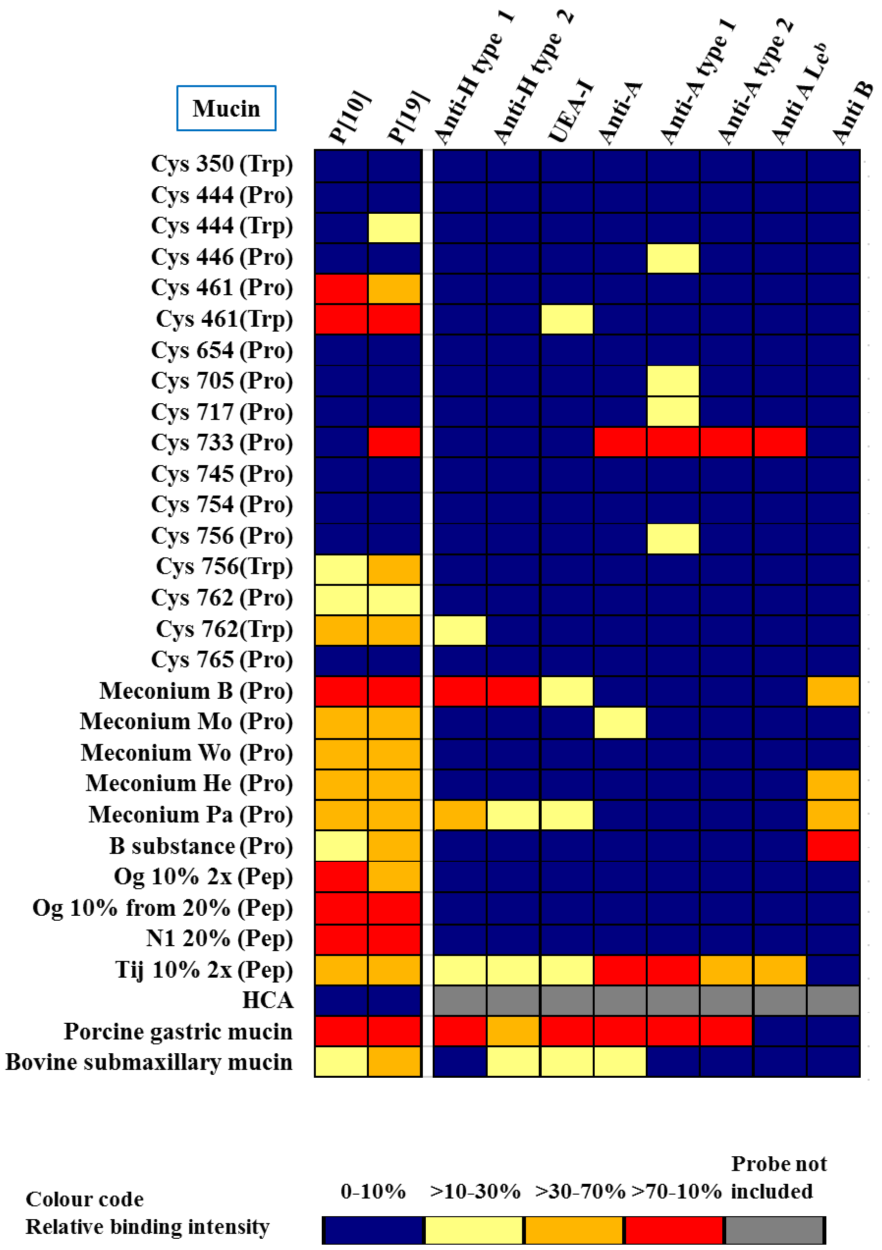

Fig. S2. Relative binding intensities of the VP8* proteins of rotavirus P[10] and P[19] (at 25 µg/mL), antibodies and the lectin *Ulex europaeus* agglutinin I in the mucin glycoprotein (primary beam search) array. The P[10] and P[19] VP8* proteins were analysed with Mucin array set 2 and antibodies and UEA-I were analysed on Mucin array set 3.

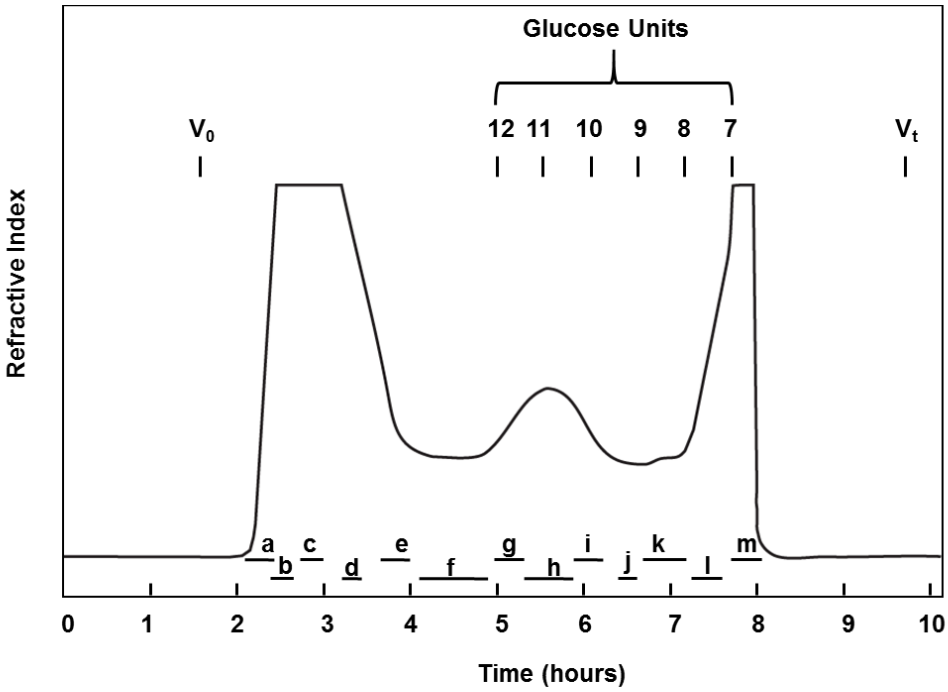

Fig. S3.| Gel filtration chromatography of the products of reductive alkaline hydrolysis from PSM. The Bio-Gel P4 column (1.6 × 90 cm) was eluted with water. V_0: void volume; V_t: total volume; numbers 7-12 at the top of the figure indicate positions of glucose units (degrees of polymerization) in a hydrolysate of dextran. Thirteen pooled fractions, a to m, were obtained.

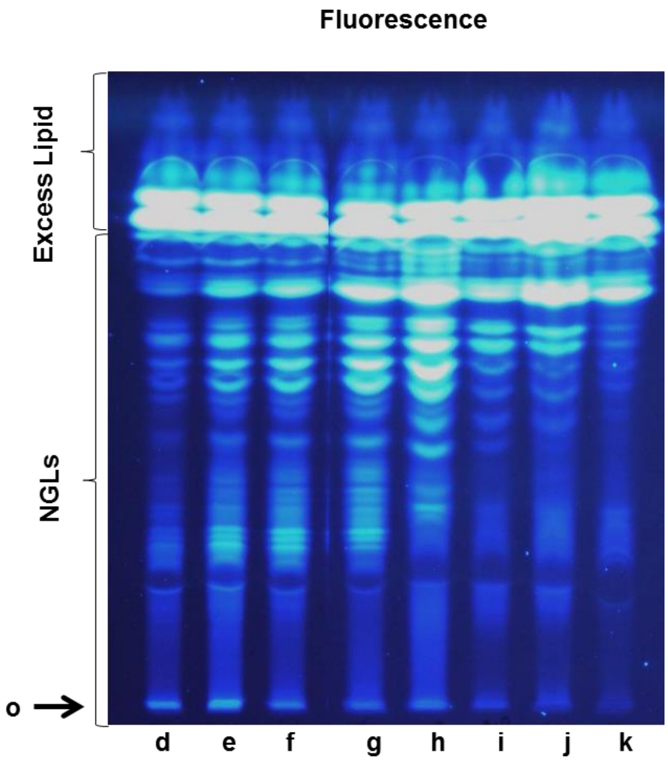

Fig. S4.| High performance TLC of the large scale NGL reaction mixtures of PSM neutral-glycan alditol fractions d to k; around 1% of the reaction volumes were applied. Solvent was $CHCl_3$/MeOH/H_2O 60:35:8.

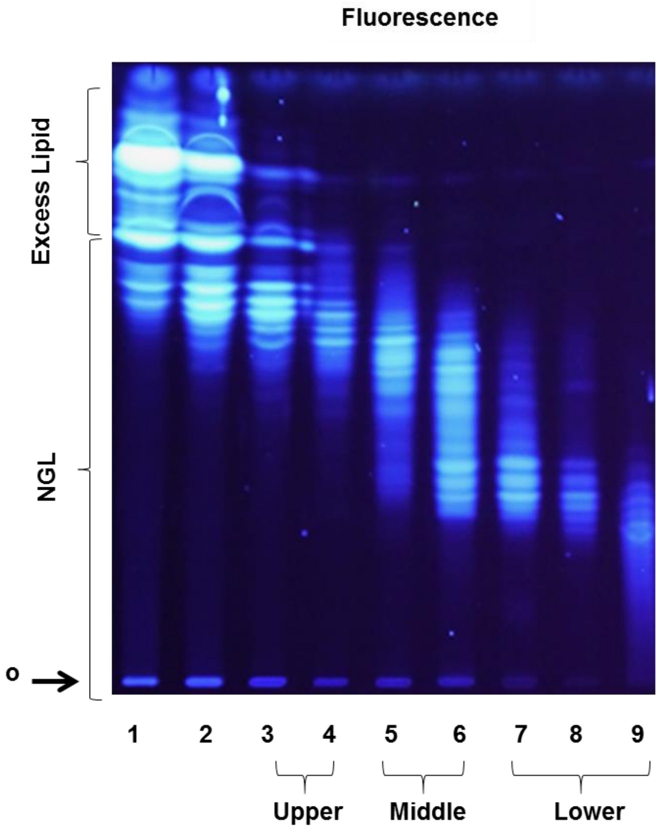

Fig. S5. High performance TLC of the pooled PSM neutral *O*-glycome NGL fractions of the reaction mixture eluted as nine fractions (4 ml each) from a short silica column; 1 µL of each were applied. The solvent was $CHCl_3$/MeOH/H_2O 60:35:8. Fractions with middle and lower migration patterns were pooled and used for generation of microarrays.

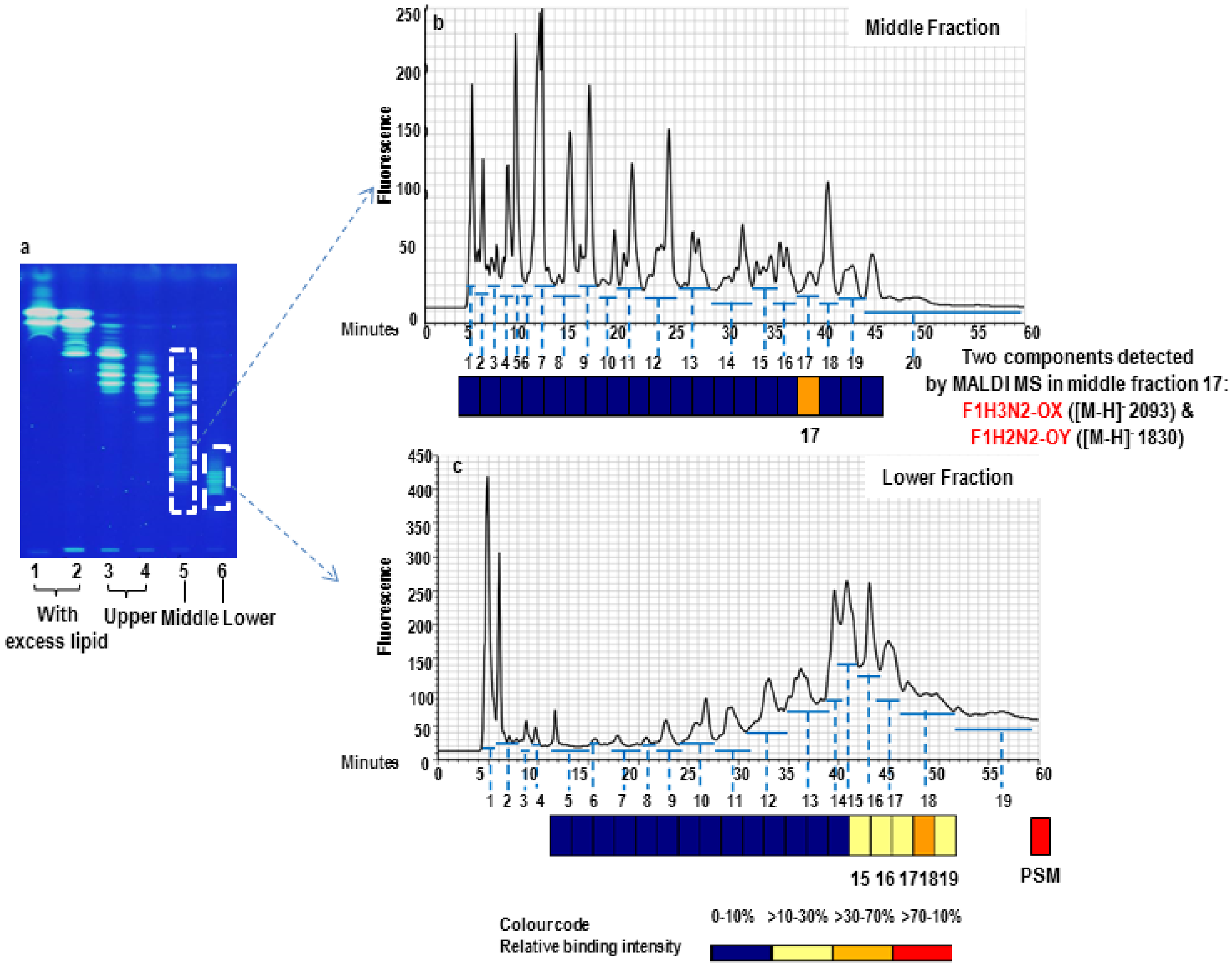

Fig. S6. Beam search array data from exploratory experiments to explore binding of the VP8* protein of rotavirus P[19] to arrayed neutral *O*-glycome NGL fractions from PSM: (**a**) high performance TLC of the pooled reaction mixture of NGLs derived from ~1mg neutral *O*-glycan alditols eluted as seven fractions from a silica column. The solvent was $CHCl_3/MeOH/H_2O$ 60:35:8. (**b**) and (**c**),Fractions with middle and lower migration patterns, respectively, were pooled as indicated and re-fractionated by HPLC. The HPLC fractions were used for generating a secondary beam search array similarly to the large scale experiments. The horizontal bars give relative intensities of binding elicited by the VP8* protein of rotavirus P[19] analysed at 50 µg/mL: 100% is for the highest signal in the array. These data are from experiments separate from those in the large scale experiment shown in **Fig. 2**. The experimental data for the small scale experiments show reproducibly of the methods used here and are available on request.

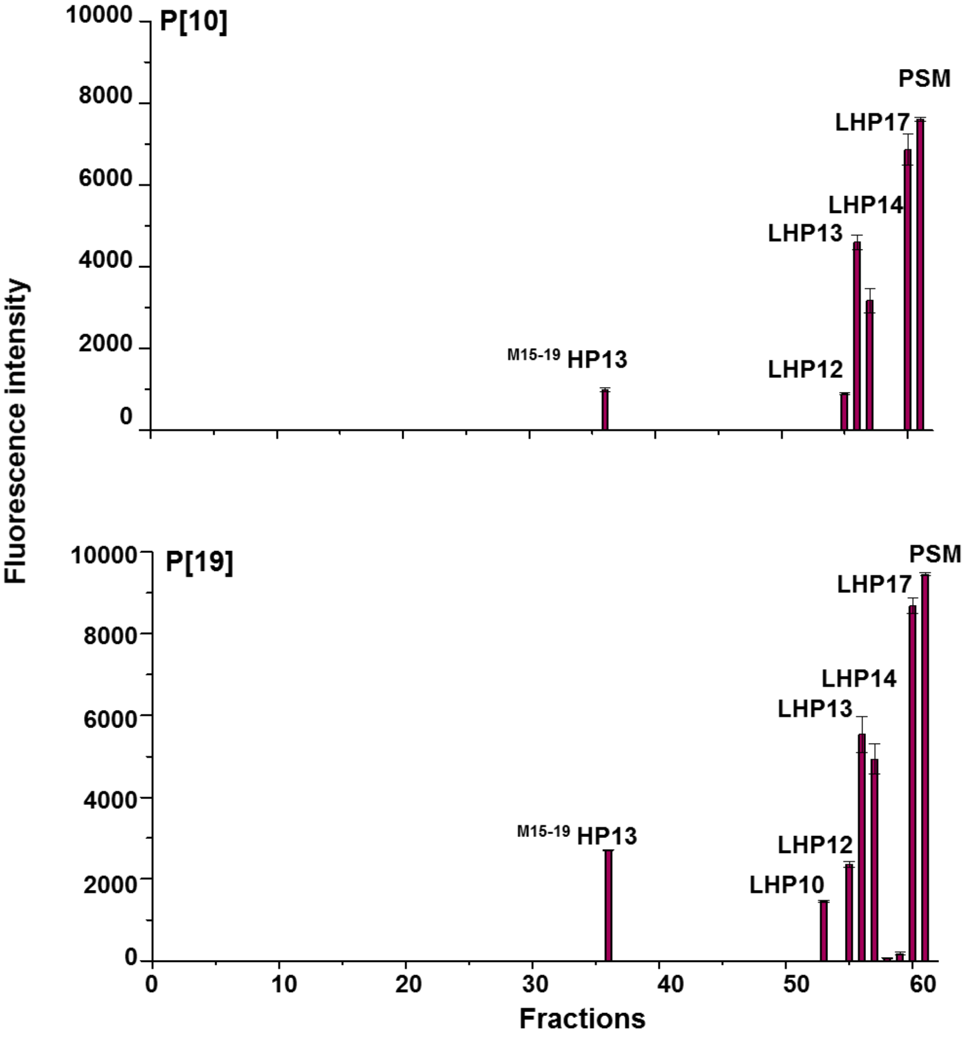

Fig. S7.| Microarray analysis of VP8* proteins of P[10] and P[19] enteroviruses using PSM neutral *O*-glycome NGL secondary beam search array. The results are the means of fluorescence intensities of duplicate spots printed at 5 fmol with error bars representing half of the difference between the two values. Both proteins were analysed at 50 µg/mL.

Appendix: Glycan Microarray Document

Based on MIRAGE Glycan Microarray Guidelines - Beilstein-Institut

Glycobiology. **2016** Nov 22. [Epub ahead of print] PMID: 27993942

**Classification**	
**1. Sample: Glycan Binding Sample**	
Description of Sample	Recombinant rotavirus VP8* proteins expressed as glutathione S-transferase (GST)-tagged proteins as described (Liu et al. J Virol 2016).<br>These and the antibodies and lectins are described in Material and Methods.
Sample modifications	no
Assay protocol	Described in Material; and Methods
**2. Glycan Library**	
Glycan description for defined glycans	Sequence defined glycan probes are described in Material and Methods and the probe names and structures are in **Tables S6, 7 and 8**. These were from the collection assembled in the course of research in the Glycosciences Laboratory (https://glycosciences.med.ic.ac.uk/glycanLibraryList.html).<br>Structure determination (by immuno-sequencing) of the hexasaccharide ligand for the VP8* protein of the two viruses are in Results section.
Glycan description for undefined glycans	The macromolecule y array of the beam search was of mucin-type glycoproteins; from among these, the ligand-positive porcine stomach mucin (PSM) was selected for generating the primary NDL array.<br><br>The neutral (non-acidic) *O*-glycans reductively released from PSM and fractionated by size were converted into fluorescent neoglycolipids (NGLs) by reductive amination following mild periodate oxidation. Sixty HPLC fractions of these NGLs were included in the PSM neutral *O*-glycome (secondary) array. Their designations, lipid contents, molecular ions and deduced compositions (from MALDI-MS analyses) are in **Table S4**.<br><br>Four sub-fractions, Bands 1-4, isolated by semi-preparative TLC from fraction $^{M15\text{-}19}$HP13 were included in the secondary array designated 'Fucase Array' (**Table S6**). Their molecular ions and deduced monosaccharide compositions are in **Figure 4b**.
Glycan modifications	Sequence defined glycan probes: Unless otherwise specified the NGLs were prepared from reducing oligosaccharides by reductive amination with the amino lipid, 1,2-dihexadecyl-sn-glycero-3-phosphoethanolamine [(DHPE) (Chai et al., Methods Enzymol. 2003)]; AO, NGLs prepared from reducing oligosaccharides by oxime ligation with an aminooxy functionalized DHPE [(AOPE) (Liu et al., Chem. Biol. 2007)].

	For full description of the lipid moieties of the glycan probes, please see https://glycosciences.med.ic.ac.uk/docs/lipids.pdf.<br>The preparation of fluorescent NGLs are described in Material and Methods.
**3. Printing Surface; e.g., Microarray Slide**	
Description of surface	Nitrocellulose-coated glass microarray slides.
Manufacturer	16-pad Slides from Sartorius Stedim, Goettingen, Germany
Custom preparation of surface	Not relevant.
Non-covalent Immobilization	The lipid-linked glycan probes were formulated as liposomes by adding carrier lipids, phosphatidylcholine and cholesterol (Liu et al., Methods Mol. Biol. 2012) for robotically arraying and non-covalent immobilization on nitrocellulose-coated glass slides. Glycoproteins were arrayed in water
**4. Arrayer (Printer)**	
Description of Arrayer	Noncontact arrayer, Nano-Plotter (Gesim, Germany)
Dispensing mechanism	Non-contact liquid delivery with four dispensing tips.
Glycan deposition	Approximate 0.33 nl was printed for each spot.<br>Each glycan probe was printed at two levels (2 and 5 fmol per spot) in duplicate. Each glycoprotein was printed at 30 and 170 pg hexose per spot
Printing conditions	The printing solutions are detailed in Online Methods
**5. Glycan Microarray with "Map"**	
Array layout	Each array slide contained 16-pad subarrays of glycan probes printed at two levels in duplicate (four spots for one probe in a row) as detailed in Material and Methods.
Glycan identification and quality control	Antibodies and plant lectins variously used to identify glycan sequences in the macromolecule array and in the primary and secondary NGL arrays are described in Material and Methods and the binding data are in **Tables S1, 5 and 6.**
**6. Detector and Data Processing**	
Scanning hardware	ProScanArray microarray scanner (PerkinElmer LAS, Beaconsfield, UK) or GenePix 4300A (Molecular Devices, Sunnyvale, USA).
Scanner settings	Scanning resolution: 10 μm / pixel (this resolution is adequate for the sizes of sample spots)<br>Laser channel: Red (scan wavelength 633 nm)<br>PMT gain 35 for ProScanArray; PMT Voltages 350V for GenePix

	Scan power: ranged from 30 to 100% to avoid saturation of binding signals
Image analysis software	ScanArray Express software (PerkinElmer) or GenePix® Pro 7 Microarray Analysis software (Molecular Devices)
Data processing	The gpr file was entered into an in-house microarray database using software (designed by Dr Mark Stoll, http://www.beilstein-institut.de/en/publications/proceedings/glyco-2009) for data processing. No particular normalization method or statistical analysis was used.
**7. Glycan Microarray Data Presentation**	
Data presentation	The microarray binding results are in **Fig 2, 3 and 4, Table 1** and in **Tables S1, 4, 5, 6, 7 and 8, and Figs 2, 6, and 7** The tables include the list of probes in the arrays, binding intensities shown are at 170 pg or 5 fmol per spot and errors (difference of signal intensities of duplicate spots of each glycan probe).
**8. Interpretation and Conclusion from Microarray Data**	
Data interpretation	No software or algorithms were used to interpret processed data.
Conclusions	1. The beam search arrays: The ligand-positive component investigated in detail is a minor component in the O-glycome of the porcine stomach mucin investigated, not previously rerported in PSM. It is assigned as a hexasaccharide with the type 1-terminating blood group H and an internal type 2 sequence, having been derived from a 3-linked branch at core GalNAcol:<br>Fucα1-2Galβ1-3GlcNAcβ1-3Galβ1-4GlcNAcβ1-Galβ-3OX<br>2. The sequence-defined arrays: corroborated the involvement of type 1-terminating blood group H in the ligand as in lacto N-fucopentaose 1 (LNF-P1)<br>Fucα1-2Galβ1-3GlcNAcβ1-3Galβ1-4Glc<br>The lower intensity of binding to LNFP-1 indiated that the backbone beyond tetrasaccharde is additionally recognized.<br>3. The complementarity of the sequence-defined arrays with the beam serch is highlighted with repect to revealing other natural glycan ligands lacking in the mucin investigated.

References

1. Watkins, W. M. (1980) Biochemistry and Genetics of the ABO, Lewis, and P blood group systems. *Adv. Hum. Genet.* 10, 1-136, 379-185
2. Hounsell, E. F., Lawson, A. M., Feeney, J., Gooi, H. C., Pickering, N. J., Stoll, M. S., Lui, S. C., and Feizi, T. (1985) Structural analysis of the O-glycosidically linked core-region oligosaccharides of human meconium glycoproteins which express oncofoetal antigens. *Eur J Biochem* 148, 367-377
3. Feizi, T., Kabat, E. A., Vicari, G., Anderson, B., and Marsh, W. L. (1971) Immunochemical studies on blood groups. XLVII. The I antigen complex--precursors in the A, B, H, Lea, and leb blood group system--hemagglutination-inhibition studies. *J Exp Med* 133, 39-52
4. Kabat, E. A. (1982) Philip Levine Award Lecture. Contributions of quantitative immunochemistry to knowledge of blood group A, B, H, Le, I and i antigens. *Am. J. Clin. Pathol.* 78, 281-292
5. Gao, C., Liu, Y., Zhang, H. T., Zhang, Y. B., Fukuda, M. N., Palma, A. S., Kozak, R. P., Childs, R. A., Nonaka, M., Li, Z., Siegel, D. L., Hanfland, P., Peehl, D. M., Chai, W. G., Greene, M. I., and Ten, F. Z. (2014) Carbohydrate Sequence of the Prostate Cancer-associated Antigen F77 Assigned by a Mucin O-Glycome Designer Array. *J. Biol. Chem.* 289, 16462-16477
6. Liu, Y., Chai, W., Childs, R. A., and Feizi, T. (2006) Preparation of neoglycolipids with ring-closed cores via chemoselective oxime-ligation for microarray analysis of carbohydrate-protein interactions. *Methods Enzymol* 415, 326-340
